# Gold(III) Complexes Activity against Multidrug-Resistant Bacteria of Veterinary Significance

**DOI:** 10.3390/antibiotics11121728

**Published:** 2022-12-01

**Authors:** Carlos Ratia, Sara Sueiro, Raquel G. Soengas, María José Iglesias, Fernando López-Ortiz, Sara María Soto

**Affiliations:** 1 ISGlobal, Hospital Clínic—Universitat de Barcelona, 08036 Barcelona, Spain; 2 Departamento de Química Orgánica e Inorgánica, Universidad de Oviedo, Julián Clavería 7, 33006 Oviedo, Spain; 3 Área de Química Orgánica, Centro de Investigación CIAIMBITAL, Universidad de Almería, 04120 Almería, Spain; 4 CIBER Enfermedades Infecciosas (CIBERINFEC), Instituto de Salud Carlos III, 28029 Madrid, Spain

**Keywords:** gold(III) complexes, antibacterial, antibiofilm, animal strains, MDR-bacteria, toxicity

## Abstract

The emergence and spread of multidrug-resistant bacteria are a global concern. The lack of new antibiotics in the pipeline points to the need for developing new strategies. In this sense, gold(III) complexes (G3Cs) could be a promising alternative due to their recently described antibacterial activity. The aim of this study was to evaluate the antimicrobial activity of G3Cs alone and in combination with colistin against pathogenic bacteria from veterinary sources. Minimal inhibitory concentration (MIC) values were determined by broth microdilution and compared with clinically relevant antibiotics. Antibiofilm activity was determined by crystal violet staining. Combinations of selected G3Cs with colistin and cytotoxicity in commercial human cell lines were evaluated. Four and seven G3Cs showed antibacterial effect against Gram-negative and Gram-positive strains, respectively, with this activity being higher among Gram-positive strains. The G3Cs showed antibiofilm activity against Gram-negative species at concentrations similar or one to four folds higher than the corresponding MICs. Combination of G3Cs with colistin showed a potential synergistic antibacterial effect reducing concentrations and toxicity of both agents. The antimicrobial and antibiofilm activity, the synergistic effect when combined with colistin and the in vitro toxicity suggest that G3Cs would provide a new therapeutic alternative against multidrug-resistant bacteria from veterinary origin.

## 1. Introduction

Among the many challenges to health, infectious diseases stand out for their ability to have a deep impact on humans and, therefore, have become a public health priority worldwide. Antimicrobial resistance (AMR) is the ability of microorganisms to become increasingly resistant to an antimicrobial to which they were previously susceptible [1,2]. Overuse and misuse of antimicrobials in both human and animal medicine has accelerated the process of the emergence and spread of antimicrobial resistant bacteria. Most antimicrobial agents used to treat human illnesses are also used in veterinary medicine [3]. There are only a few classes of antibiotics exclusive to humans, such as carbapenems [4]. Scientists have expressed their concerns that the use of these antibiotics in animals may lead to a decrease in their effectiveness on human medicine due to the selection of resistant bacteria. The transmission of these resistant bacteria can occur by direct contact with the animals, through the food chain, or through the contact with contaminated resources related to farming [3]. Colistin (CST) illustrates an important example of this fact. It belongs to the polymyxin class of antimicrobials and acts at the bacterial cell membrane level. Due to its high toxicity and the availability of other safer and more effective therapies, CST was taken out of clinical use and restricted to the veterinary sector [5]. However, the emergence of MDR bacteria together with the lack of availability of new antibiotics has led to its return in clinical practice [4]. Resistance in humans was reported after colistin was reintroduced [6]. The extensive use of CST in animals was recognized as the agent responsible for the emergence and spread of these mechanisms, demonstrating that animals can be reservoirs of antibiotic resistance genes (ARGs) and can transfer them to humans [7,8].

As microbes are ubiquitous, addressing this health threat only from the human perspective would lead us to underestimate its actual reach. A critical juncture between humans and animals is found in zoonotic diseases. Bacteria and their genetic material can easily disseminate among humans, animals, and the environment. For this reason, emphasis should be placed on the term One Health, which embodies the principle that human and animal health are interconnected, and with the environment, and should be addressed as a whole [1,9]. This approach is essential to develop supportive measures to tackle AMR.

The AMR crisis among clinically important pathogens together with the lack of therapeutic drugs, supports the idea that new strategies with novel mechanisms of action are needed. Since 2017, eleven new antibacterial agents have been approved, but only two contain a new active substance (vaborbactam and cefiderocol), the remaining were reformulations of existing classes [2]. This context is exacerbated by the withdrawal of large pharmaceutical companies from the antibiotic market over the past 20 years. The scientific difficulties for the development of new drugs, the requirements of governments and regulatory agencies, and the poor financial returns associated with antibiotic development have led to this scenario [10]. New approaches are urgently required to find antibiotics with different target mechanisms from the current ones and metal-based drugs may be an important source of research [11].

The use of structurally defined metal complexes in medicine is not new. Since the beginning of the 20th century, several organometallic compounds have been used for the treatment of several pathologies [12,13,14]. In this regard, gold-based compounds have attracted much attention in medicinal chemistry over the last 10 years, leading to promising metallodrugs with applications mainly in the treatment of cancer [15]. However, even though the first studies evidencing the antimicrobial activity of gold complexes date back to Robert Koch’s studies at the end of 19th century [16], it was only recently that the use of gold gained attention for the development of metalloantibiotics [17].

Considering that gold(III) complexes are well-known as cytotoxic agents, a balance between cytotoxicity and antimicrobial activity is crucial for the development of new gold(III)-based metalloantibiotics [11]. Despite many of the reported gold(III) complexes having a selectivity index in principle (that is, higher IC_50_ values for the antiproliferative activity against cancer and human cells than the MIC values against bacteria), the therapeutic index usually needs to be increased by structural optimization [18,19].

We previously developed a novel (S^C)-cyclometallated gold(III) complex **1** based on an *ortho*-substituted phosphinothioic amide [20,21]. The interesting antibacterial profile of complex **1** prompted additional studies. Thus, in order to further stabilize the gold(III) centre and increase the metabolic stability, the chlorine atoms were substituted by a bidentate dithiocarbamate (DTC) ligand, dramatically improving not only the stability but also the antibacterial activity. Thus, these cationic mixed cyclometallated (C^S)-Au(III)/DTC complexes displayed antimicrobial activities against a broad range of bacterial strains belonging to different Gram-positive and Gram-negative species [22].

**Figure 1:**
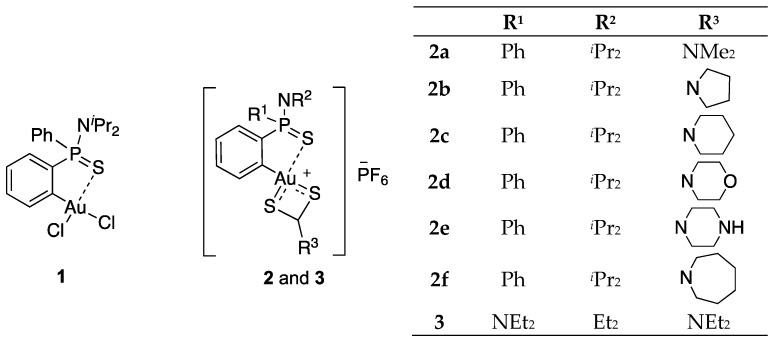
Structure of the complexes included in the study.

In view of the AMR challenge together with the lack of effective treatments against planktonic and biofilm pathogenic bacteria of either clinical or veterinary origin, synthetic compounds such as gold(III) complexes could be a promising therapeutic alternative. In this context, we decided to expand the investigation on (C^S)-cyclometallated Au(III) complexes and selected eight representative molecules—C^S and S^S bidentate ligands—for antibacterial screening (compounds **1**–**3**, Figure 1). The selection was initially performed based on previous results on the antibacterial activities of the compounds in pathogenic bacteria of clinical human origin (data not published) and by the structural differences in the (S^S)-DTC ligand (compounds **2a**–**2b**) and the (C^S)-cyclometallated ligand (compound **3**). In this regard, the complexes with the most promising antimicrobial activities against both Gram-positive and Gram-negative strains were chosen for further evaluation on strains of animal origin.

## 2. Results

### 2.1. Antibacterial Activity on Planktonic Bacteria

Minimal inhibitory concentration values are compiled in Table 1. We observed that all gold(III) complexes presented higher antibacterial activity among Gram-positive strains than the Gram-negative counterparts. Thus, except for complex **1**, the MIC values ranged from 0.03 to 1 mg/L among almost all the different studied Gram-positive strains. Only *S. uberis* presented MICs of 2 or 8 mg/L for **2a**, **2b** and **2c**, respectively. The MIC values obtained for gold complex **1** against Gram-positive strains were similar to those obtained against human strains [22]. It should be noted that gold(III) dithiocarbamate complexes were more active against Gram-positive strains than the comparative antibiotics, ampicillin (except in the case of *S. xylosus*) and vancomycin (VAN). In the case of Gram-negative species, and similar to what was observed with human strains [21], **1** did not show antibacterial activity, observing MIC values equal to 64 mg/L. Among the gold(III) complexes studied, **2f** was the most active with MICs of 4–8 mg/L from all Gram-negative species including *P. aeruginosa*. Complexes **2a**–**2d** showed moderate antibacterial activity against Gram-negative strains with MIC values ranging from 8 to 16 mg/L in most cases. However, complex **2a** was the most active complex against Gram-positive strains. Complex **2e** displayed low activity against Gram-negative strains and good activity against Gram-positive strains except in the case of *S. uberis*. On the other hand, **2b** and **3** presented similar behavior, showing similar MIC against *Salmonella* spp., *E. coli* and *P. aeruginosa* strains. We observed that in each Gram-negative species, MIC values for the same molecule tend to remain constant, with variations of ± one-fold (Table 1). All the gold(III) complexes were more active than ampicillin and showed similar performance to trimethoprim-sulfamethoxazole but less than colistin and ciprofloxacin.

In order to know the bactericidal or bacteriostatic behavior of the different molecules, the minimal bactericidal concentrations (MBCs) were determined. Thus, among the Gram-negative strains, the MBC values were one to two-fold higher than the MICs (Table 2). It is remarkable that in the case of the *E. coli* 2642 ME-2.2 and GN444, the MIC and MBC of **2a**–**2d** presented the same value.

In the case of *S. chromogenes* strains, the MBC values were very similar to the MIC values, that is, they were equal or lower than 1 mg/L with all the gold(III) complexes except from those obtained with complex **1**. However, the MBC values for *S. uberis* were higher than 8 mg/L, more than three-fold the MIC (Table 2).

### 2.2. Assessment of Biofilm Formation

**Figure 2:**
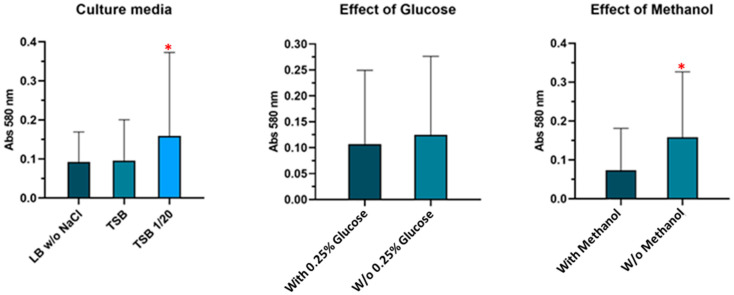
Conditions tested for the induction of biofilm formation in *Salmonella* spp. using the three strains under study. Data shown are mean values of three replicates and standard deviations. Red asterisks (*) indicate significant differences (*p* < 0.05) between conditions.

Standardization of experimental conditions was initially carried out to induce and detect biofilm formation among *Salmonella* strains. The conditions tested for were different culture media, the effect of glucose in the medium and prefixation with methanol. A higher and statistically significant (*p* < 0.05) biofilm formation was observed when 1/20 Tryptic Soy Broth (TSB) without 0.25% glucose, and prefixation of biofilm with methanol was used (Figure 2).

**Figure 3:**
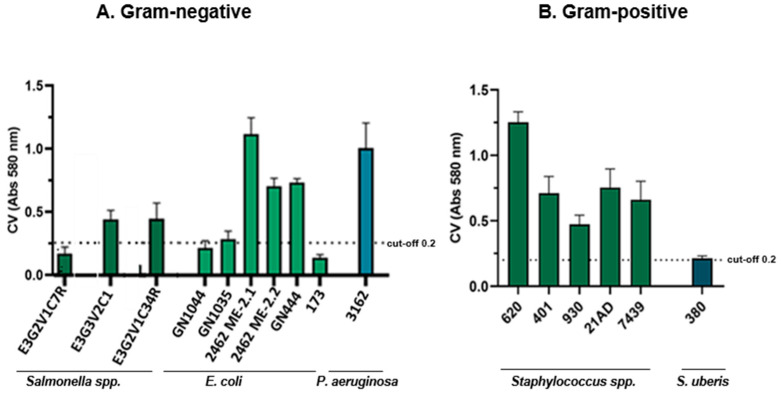
Results obtained after CV reading at 580 nm to identify biofilm-forming strains. Mean values of three readings are represented. (**A**) Gram negative strains. (**B**) Gram-positive bacteria.

Once we had the experimental protocols and conditions, we carried out the induction and detection of biofilm formation for each strain. Thus, after absorbance reading at 580 nm, the isolates were grouped according to the cut-off value of 0.2. As is illustrated in Figure 3, among the 16 strains evaluated, eight were biofilm-forming, including five Gram-negative and three Gram-positive bacteria. *E. coli* 2462 ME-2.1 and *S. haemolyticus* 620DD1 were classified as strong biofilm formers with an optical density (OD) of 0.871 and 1.252, respectively. Five strains were classified as weak biofilm producers, five as moderate biofilm producers, and four as no-biofilm formers.

### 2.3. Antibiofilm Activity

On the basis of the results achieved in the identification of biofilm-forming strains, representative strains of each species were selected to evaluate antibiofilm activity of gold(III) complexes. CST and VAN were used as controls in the case of Gram-negative and Gram-positive strains, respectively.

Results showed that three gold(III) complexes exhibited high antibiofilm activity against *E. coli* strains: **2b**, **2c**, and **2f** (Table 3) with minimal biofilm inhibitory concentrations (MBICs) ranging between 4 and 8 mg/L. Among *E. coli* strains, **1**, **2a** and **2d** presented MBIC values lower than the corresponding MICs although only one to two-fold of difference. Complex **1** showed an MBIC of 16 mg/L that was lower than those obtained among human clinical strains [21]. In the case of **2b**, **2e** and **2f**, MBIC values were very similar to the MIC among *E. coli* and *S. panama* strains.

Surprisingly, and taking into account the high antibacterial activity of the studied gold(III) complexes against Gram-positive strains, none of the complexes were able to inhibit biofilm formation among the Gram-positive forming biofilm, showing MBICs higher than 32 mg/L (Table 3).

### 2.4. Analysis of Antimicrobial Synergy

As we previously observed a synergistic behavior of gold(III) complexes when they were used in combination with CST against Gram-negative human bacteria [22], we tested combinations of **2b** with CST against the only CST resistant strain, *E. coli* GN1044.

Complex **2b** plus CST showed a synergistic effect with fractional inhibitory concentration index (FICI) values of 0.375. It is especially remarkable that, in combination, the concentration of **2b** necessary to inhibit growth is three-fold lower than in monotherapy (from 16 to 2 mg/L), and the MIC of CST decreased from 32 mg/L in monotherapy to 4 mg/L.

### 2.5. In Vitro Cytotoxicity Assessment

**Figure 4:**
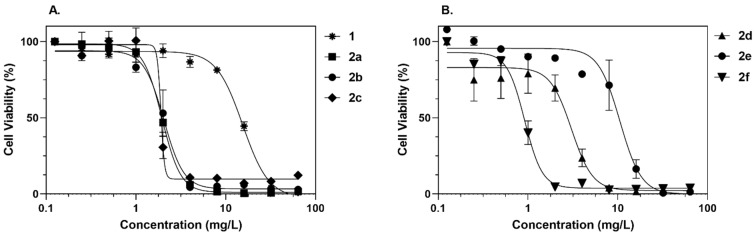
Cytotoxicity of the gold(III) complexes **1**, **2a**, **2b**, **2c** (**A**) and **2e**, **2d**, **2f** (**B**) on Jurkat E6.1 cells. Mean and SD of three experiments are represented in the graph.

Considering the potential of gold(III) complexes as therapeutic antimicrobial agents, we evaluated the in vitro toxicity profile of all molecules under study in the human tumoral Jurkat E6.1 cell line. Figure 4 shows the in vitro cytotoxicity results in a dose-response curve. We can observe that the gold(III) complexes **1** and **2e** showed IC_50_ values of 18.74 mg/L and 10.53 mg/L, respectively. Whereas the toxicity profile for **2c**, **2d**, and **2f** were less favorable with values of 6.353 mg/L, 4.206 mg/L and 3.521 mg/L, respectively. DMSO, used as a control, reported an IC_50_ > 64 mg/L, which implies that the toxicity generated by any of the gold(III) complexes is not influenced by the presence of this solvent.

## 3. Discussion

Antibiotic resistance crisis is widespread and worsening, due to the continued overuse of antibiotics and the failure of industry to develop new antibiotics [2]. Infections with MDR bacteria are compromising global health, threatening the effectiveness of antibiotics, and driving the emergence of resistance not only among the clinical setting but also among animals and the environment [23]. Not only the emergence and spread of antibiotic resistant microorganisms among animals have important consequences on public health but also negative impact on animal health and welfare itself. Thus, the high use in veterinary services and as growth-promoter on animals for food production has left a reduction of the number of antibiotics that are effective to treat several infectious diseases. Therefore, if animals directed to food production cannot be treated for an infectious disease, the impact will be not only in the animal health but also on productivity and economy [24].

To face this health challenge, new therapeutic alternatives with different target mechanisms are beginning to be investigated, where metal-based drugs are an attractive source of research [25]. In this work, we focused on gold-based compounds due to their promising antimicrobial potency and set out to evaluate the antimicrobial activity of C^S-thiophosphinamide- and C^S-thiophosphonamide-dithiocarbamate gold(III) complexes against pathogenic bacteria from veterinary sources.

The antimicrobial activity recorded in the planktonic assays showed four gold(III) complexes potentially active against the Gram-negative species *Salmonella* spp., *E. coli*, and *P. aeruginosa*, as well as seven gold(III) complexes against the Gram-positive species of *Staphylococcus* spp. and *S. uberis*. Not only do we observe differences in the number of active gold(III) complexes between these two bacterial groups, but there is also a reduction in MIC values for Gram-positive microorganisms. The difference displayed among Gram-negative and Gram-positive bacteria could be explained by their architecture. The outer membrane of Gram-negative bacteria is the main reason for resistance to a wide range of antibiotics, as most antimicrobials must pass through it to reach their targets. In contrast, Gram-positive bacteria lack an outer membrane, making Gram-negative significantly more resistant to antibiotics than Gram-positive bacteria [26]. Previous studies on the antimicrobial activity of analogous gold(I) and gold(III) complexes showed a broad spectrum of antibacterial activities, and some even had a certain degree of selectivity towards Gram-positive species [27]. This trend is similar to that observed among clinical bacterial strains using the gold(III) complex **1**, presenting the lowest MIC values (8–16 mg/L) among the Gram-positive strains in contrast to Gram-negative strains (64 mg/L) [21]. Taking into account that *Staphylococcus* spp. and *S. uberis* are among the main causative agents of animal mastitis, the activity of our molecules seems to be very promising against these bacterial species. In the case of *S. uberis*, high levels of resistance to aminoglycosides, aminocoumarin, and novobiocin have been reported [28].

Our results have demonstrated that inhibitory and bactericidal concentrations of gold(III) complexes tended to remain constant within the different isolates of the same species. Concerning the origin, the activity of gold(III) complexes seems to be similar among human clinical or veterinary isolates. Thus, except in the case of **2b**, **2d** and **3**, which were more active against human clinical strains, the MICs observed in this study for the veterinary strains are in concordance with previous data obtained by the research group against human clinical isolates from the same species (unpublished data). 

The tested concentrations of the gold(III) complexes have reflected a bacteriostatic effect on bacterial growth of both Gram-negative and Gram-positive bacteria, although we have also observed that the increase of one or two folds in these concentrations leads to a bactericidal effect, except for **2f** which presents a greater increase for any of the species. As mentioned above, the overuse of antibiotics has led to the emergence of bacteria that can withstand inhibitory concentrations and can prompt bacteria to persist [23], which is why the emphasis should be focus on development of antibiotics with bactericidal effect.

The impact of biofilms on human infections has been studied more in depth than in animal health, having a high importance in veterinary medicine, leading to a negative repercussion in economy and animal production. This biofilm state of life brings to bacteria a high resistance to antibiotics and immune system. The biofilm structure confers resistance via multiple mechanisms: delayed diffusion through the polysaccharide matrix; expression or repression of different genes that confer a range of physiological responses; and growth rate reduction and presence of persister cells [29].

Currently, the trend on biofilm treatment in animals is to use combinations of antibacterial and antibiofilm agents, electric currents, nanocarriers and ultrasound [30]. Thus, disruption of the biofilm would facilitate the efficacy of antimicrobial agents and minimize the scope for the development of antibiotic resistance [31]. The investigated gold(III) complexes affected biofilm formation among all Gram-negative strains studied, obtaining the same value or ±1-fold compared to their planktonic activity. However, against *Staphylococcus* spp. no biofilm inhibition at the tested concentrations was observed. According to our results, antibiofilm activity of several gold(III) complexes has been shown against *P. aeruginosa* in either pre-adhesion and post-adhesion phases of the biofilm formation [27]. Nevertheless, there are studies of metal-based drugs such as gold-complexed sulfonamide that effectively promoted a remarkable reduction in the bacterial adhesion against *S. aureus* strains [32].

CST is one of the antibiotics that has been used in veterinary medicine for decades to prevent and treat infections caused by Enterobacteriaceae species. This antibiotic has been widely used in animal feed with the consequence that it can reach humans through the food chain [33]. In humans, it is considered a last resort antibiotic due to its neurotoxicity and nephrotoxicity [34]. However, colistin has been recovered to treat those MDR bacterial strains that conventional antibiotics are not able to kill. When monotherapy treatment of both CST and **2b** was compared with the combined action of **2b** gold(III) complexes with CST, the activities of the single agents were enhanced and a synergistic effect was achieved in the trials. The mechanism of action of CST is based on binding to lipopolysaccharides and phospholipids of the outer cell membrane of Gram-negative bacteria [35]. *E. coli* GN1044 were resistant to CST, but the combined action of these antimicrobials was able to inhibit bacterial growth by reducing the concentrations of each agent at least three-fold. In fact, the combined action with the gold(III) complex **2b** was able to lower the MIC of CST below the breakpoint of ≤2 mg/L, according to EUCAST guidelines [36], same values as those found in bacteria susceptible to this antibiotic. Similar results were found using **1** against CST-resistant clinical strains, decreasing four-fold the MIC of CST and **1** [21]. Studies have shown the synergistic effect of CST in combination with other clinically relevant antibiotics such as ceftazidime against MDR bacteria [37]. Researchers have proposed that CST may exert a subinhibitory permeabilizing effect on the outer membrane of Gram-negative bacteria even in CST-resistant isolates [38]. It appears that the outer membrane compromises the activity of gold(III) complexes, so that when CST permeabilizes the bacterial outer membrane, the activity of gold(III) complexes is greatly enhanced. Indeed, this provides a promising therapeutic approach for animals infected with CST-resistant Gram-negative pathogens. A plausible explanation for the observed synergy between the gold complexes and colistin would be in the initial binding of the latter to anionic lipid A molecules of Gram-negative bacteria [5,6], leading to a rapid permeabilization of the outer cell membrane that allows improved penetration of the gold(III) complexes [39,40]. In the absence of colistin, the permeability barrier conferred by the outer membrane of Gram-negative bacteria impairs the transfer of gold complexes to the cytoplasm [17], so that when CST permeabilizes the bacterial outer membrane, the activity of gold(III) complexes is greatly enhanced. Indeed, this provides a promising therapeutic approach for animals infected with CST-resistant Gram-negative pathogens.

Even though the data set is very limited, it is possible to gather some useful information regarding the SAR. In general terms, Au(III) (C^S)-cyclometallated complexes **2** and **3** with a bidentate (S^S) ancillary ligand are more active against both Gram-positive and Gram-negative strains than complex **1**, containing two monodentate chloro ancillary ligands. Regarding the structure of the cyclometallated core, there are not salient differences in the activity of dppta complexes **2** when compared to bppta complex **3**.

Complexes **2** and **3** are all very active against Gram-positive strains and the best results were obtained for complex dimethyldithiocarbamate **2a**. In this sense, complex **2a** is the less sterically hindered of the whole series, which could contribute to its higher effectiveness, because the lack of bulky substituents facilitates intermolecular interactions between the complexes and molecular components of target cells. Along this hypothesized steric effect is the excellent activity of complex **3** against Gram-positive strains. However, the very good activity of morpholinyl dithiocarbamate **2d** is rather surprising considering steric effects alone.

Steric factors also cannot explain the pattern observed for the activity of DTC complexes **2** and **3** against Gram-negative strains. In this case, complexes **2b**, **2c** and **2f** containing cyclic aliphatic ligands display better antibacterial activity than complexes **2a** and **3** with smaller ligands. Moreover, the best activity in the series is observed for complex **2f** with the largest azepanyl dithiocarbamate ligand. These results point to a different mechanism of antibacterial action for Gram-negative strains; a plausible hypothesis would be the intercalation of multiple molecules of the gold complex within the bilipid layer causing membrane damage, an effect which was previously described for the action of gold complexes on Gram-negative bacteria [41]. This intercalation would be more effective with bigger complexes, explaining the higher activity of the most sterically hindered complex.

Regarding the antibiofilm activity, biofilm is a complex matrix of extracellular polymeric substances, and unlike planktonic cells, it has a strongly hydrophobic character [42]. Therefore, biofilm would have more affinity to apolar substances, which would explain the greater effectiveness in the treatment of the biofilm of more apolar complexes **2b**, **2c** and **2f**.

Furthermore, gold(III)-complex **1** showed lower in vitro toxicity in the Jurkat E6.1 cell line compared to the DTC complexes **2a**–**f** and **3** panel of molecules tested, which is consistent with the results of previous studies [22]. Specifically, this gold(III) complex showed lower antibacterial activity against Gram-negative cells than **2a**, **2b** and **2e** which had been classified as potential antimicrobial and antibiofilm agents. The slightly increased toxicity over Jurkat E6.1 cells (a tumoral cell line) could be related to the antitumoral activity commonly associated with gold(III) complexes [43]. In contrast to Gram-positive bacteria, the three gold(III) complexes with the lowest cytotoxicity at therapeutic concentrations are classified as active molecules in this group. Some studies suggested that the cytotoxicity of gold(III) complexes is associated with the presence of the gold(III) center [44].

## 4. Materials and Methods

### 4.1. Chemistry

The target (C^S)-cyclometallated Au(III) complexes were synthesized using established procedures with minor modifications. Thus, [Au(dppta)Cl_2_] complex **1** (dppta = *ortho*-*N,N*-diisopropyl-*P,P*-diphenylphosphinothioic amide) was prepared through tin(IV)-gold(III) transmetalation from the corresponding chlorodimethylstannyl derivative as previously reported [20].

[Au(dppta)(dtc)][PF_6_] complexes **2a**–**f** (dtc = dithiocarbamate, R_2_NCS_2_) were synthesized by the overnight reaction of [Au(dppta)Cl_2_] complex **1** with the corresponding dithiocarbamate sodium or potassium salt [45] in methanol, followed by the addition of aqueous potassium hexafluorophosphate (Method M1, Supplementary Materials).

The products were characterized by ^1^H, ^13^C and ^31^P NMR, IR and HRMS. The transformation of the [Au(dppta)Cl_2_] complex **1** to the [Au(dppta)(dtc)][PF_6_] complexes was confirmed by the upfield shift in the characteristic signal of the H *ortho* to the Au (from δ_H_ H 8.47 to 7.75 ppm for **2a** and **2b**; 8.01 ppm for **2c**; 7.84–7.91 ppm for **2d** and **2e**; 7.74–7.80 ppm for **2f**) in the ^1^H NMR (Figures S1, S4, S7 and S10, Supplementary Materials). On the other hand, the ^31^P NMR spectra of complex **2** display a significant downfield shift from δ_P_ 68.2 (**1**) to 75.5–76.5 ppm (Figures S3, S6, S9 and S12, Supplementary Materials). The ^13^C NMR resonance for the NCS_2_ carbon appeared between δ_C_ 190.6 and 198.6 ppm (Figures S2, S5, S8 and S11, Supplementary Materials). Bands in the IR spectra between 1528 and 1579 cm^−1^ are attributed to the delocalized dithiocarbamate NCS_2_ system, while bands around 837–840 cm^−1^ are due to the PF_6−_ anion. The complexation of the DTC ligand is further supported by the ion [M]^+^ observed in mass spectrometry for complex **2**. The lack of fragmentation indicates appreciable stability, and is in agreement with other gold(III) complexes containing bidentate ligands [46].

Similarly, addition of sodium diethyl dithiocarbamate to a methanolic solution of [Au(bppta)Cl_2_] complex **4** (bppta = *ortho*-bis(*N,N*-diethyl)-*P*-phenylphosphonothioic amide, [47] afforded, after addition of aqueous potassium hexafluorophosphate, [Au(bppta)(Et_2_NCS_2_)][PF_6_] complex **3** (Method M2, Supplementary Materials, δ_P_ 68.2, Figure S15).

The transformation of the [Au(bppta)Cl_2_] complex **4** to the [Au(bppta)(Et_2_NCS_2_)][PF_6_] complex **3** was confirmed by the upfield shift of the signal of the H *ortho* to the Au [Au(bppta)Cl_2_] complex **4** from δ_H_ 8.24 to 7.48 ppm in the ^1^H NMR (Figure S13, Supplementary Materials). On the other hand, the ^13^C NMR spectrum displays the resonance for the NCS_2_ carbon at δ_C_ 195.1 ppm (Figure S14, Supplementary Materials). Further confirmation of the presence of the dithiocarbamate arises from the band observed at 1552 cm^−1^ in the IR spectra due to the C-N stretching absorption of the NCS_2_ system. The complexation of the dtc ligand is further supported by the ion of *m/z* 629.1323 observed in mass spectrometry corresponding to the cation [M]^+^ (calcd. for C_19_H_33_AuN_2_PS_3_ *m*/*z* 629.1313). The HRMS supports the formation of [Au(bppta)(Et_2_NCS_2_)][PF_6_] complex **3**, showing a peak for ion [M]^+^. Again, the absence of fragmentation is indicative of a good stability.

More detailed information about the experimental procedures and full spectroscopic for new complexes are included in the Supplementary Materials (Sections S1 and S3, respectively). Stock solutions were prepared in 100% dimethyl sulfoxide (DMSO, Sigma-Aldrich, Madrid, Spain) at 3000 or 6000 mg/L and stored at −20 °C until use. The final concentration used in the biological experiments was 0.5%, without exhibiting toxic effects.

### 4.2. Bacterial Strains

A panel of 16 strains of animal origin were used for the study: three *Salmonella* spp., six *E. coli*, five *Staphylococcus* spp. from different species, one *S. uberis* and one *P. aeruginosa*. These isolates came from the Institute of Agrifood Research and Technology (IRTA) of Barcelona (Spain). MALDI-TOF mass spectrometry was used to confirm identification and the strains were subsequently stored at −80 °C in skim milk (Becton Dickinson, Barcelona, Spain). *E. coli* ATCC 25922, *P. aeruginosa* ATCC 27853, and *Staphylococcus aureus* ATCC 29213 reference strains were included as controls.

### 4.3. Minimal Inhibitory Concentration Determination

MICs were determined using the broth microdilution method, following the CSLI guidelines [48]. The tests were conducted in ISO-Sensitest Broth (Oxoid, Hamsphire, UK), according to recommendations of the International Standard ISO 20776-1. Standard antibiotics were also included in the assays in order to compare their activity and efficacy. In particular, ciprofloxacin, ampicillin, vancomycin, trimethoprim-sulfamethoxazole (Sigma Aldrich, Madrid, Spain), and colistin (MPBiomedicals, Eschwege, Germany) were investigated.

Briefly, broth microdilution was performed in polystyrene 96-well round bottomed plates in a final volume of 100 μL. Serial two-fold dilutions of the antimicrobial ranging from 128 mg/L to 0.03 mg/L were used. In addition, wells with bacteria alone and with growth media alone were included as positive and negative controls, respectively. As for the inoculum, after an overnight incubation in agar plates, bacterial colonies were resuspended in 0.9% NaCl to reach a 0.5 McFarland (equivalent to 1.5 × 10^8^ CFU/mL) and diluted to obtain a final concentration in the well of 5 × 10^5^ CFU/mL. Finally, the plates were incubated for 18 h at 37 °C. MIC values were expressed in mg/L. We performed three technical and three biological replicates for each agent.

### 4.4. Minimal Bactericidal Concentration Determination

The MBCs are known as the lowest concentration of an antimicrobial agent that will prevent the growth of 99.9% of bacteria [49]. This is done by subculturing the broth dilutions used for MIC determination (i.e., wells above the MIC) on fresh agar plates [50]. Serial two-fold dilutions of the antimicrobial ranging from 128 mg/L to 0.03 mg/L were used. Thus, after microdilution assays, 10 μL aliquots of all wells showing no visible pellets or turbidity were seeded onto Luria-Bertani (LB) plates and incubated for 24 h at 37 °C. Results were expressed in mg/L and MBC values were defined as the last well at which no regrowth was observed.

### 4.5. Biofilm Formation

Biofilm formation standardization was carried out only for microorganisms without previous experimental protocols in the laboratory, specifically for *Salmonella* spp. strains. The main aim was to evaluate the best experimental conditions and requirements for detecting and inducing large amounts of biofilm in these strains. The possibilities studied were different for both species and were chosen after reviewing the literature.

For *Salmonella* spp., this study was carried out with three different strains. We tested three culture media: TSB diluted 1/20, and Luria Bertani broth (LB). Additionally, each medium was screened supplemented with or without 0.25% glucose (Merck Millipore, Bedford, MA, US). The last condition tried was a pre-fixation step with 100 μL of methanol (VWR Chemicals, Barcelona, Spain) during 15 min before quantification, as a way to attach the film [51,52,53,54].

Prior to analysis, tested strains were cultivated on solid medium and transferred into liquid cultures. These bacterial suspensions were made with LB broth (Condalab, Barcelona, Spain) for Gram-negative strains and TSB (Condalab, Spain) for Gram-positive strains. Incubation was carried out overnight at 37 °C with shaking. Overnight cultures in LB were diluted into the corresponding media and the turbidity was adjusted to match 1.5 × 10^8^ CFU/mL (McFarland 0.5) [52]. Plates were filled with 75 μL of the suitable medium and 75 μL of bacterial suspension. Negative control wells containing only broth were included. The inoculated plate was covered with a lid and incubated aerobically 48 h at 37 °C, with the exception of *E. coli* which was incubated at 30 °C. The planktonic suspension was carefully removed. Then, 150 μL of 1x phosphate-buffered saline (PBS) (Merck Chemicals, Bedford, MA, USA) was added for washing. The content was again poured off in order to remove all non-adherent bacteria and plates were dried for 15–20 min at 65 °C. Afterwards, microtiter plates were stained 10 min with 150 μL of 1% crystal violet (CV) (Comercial Bellés, Tarragona, Spain) per well. Excess dye was vigorously shaken and washed with 150 μL of 1× PBS. Later, empty plates were dried 20–25 min at 65 °C. The attached biofilm stained with crystal violet was dissolved in 100 μL of 33% glacial acetic acid (Panreac, Castellar del Vallés, Spain).

Results were obtained by measuring the OD of each well at 580 nm using an Epoch™ Microplate Spectrophotometer (BioTek, Winooski, VT, USA). Average OD values were calculated for all tested bacteria and negative controls. Interpretation of the results was achieved by using a cut-off value of 0.2 (ODc) in order to separate the strains in different categories: OD ≤ ODc = no biofilm producer; ODc < OD ≤ 2× ODc = weak biofilm producer; 2× ODc < OD ≤ 4× ODc = moderate biofilm producer; and 4× ODc < OD = strong biofilm producer [55].

### 4.6. Antibiofilm Activity

The biofilm inhibitory activity of the gold(III) complexes was determined against the biofilm-forming strains included in the study. Serial two-fold dilutions of each gold(III)-complex, ranged from 64 to 0.5 mg/L, were performed in flat-bottomed 96-well microtiter plates with the corresponding culture media to enhance biofilm formation (TSB diluted 1/20 for *Salmonella* spp., M63 supplemented with 0.25% glucose for *E. coli*, LB supplemented with 0.25% glucose for *P. aeruginosa*, TSB with 0.25% glucose for *Staphylococcus* spp., and TSB with 5% horse-lysed blood for *S. uberis*). Bacterial suspensions were prepared from overnight cultures, and the turbidity was adjusted to a 0.5 McFarland in the corresponding media for each species and consequently diluted to reach an inoculum of 5 × 10^6^ CFU/well. For *P. aeruginosa*, biofilms were formed by immersing pegs of a modified polystyrene microtiter lid (Nunc TSP System, Nunc, Rockslide, Denmark). After 48 h incubation at 37 °C in static, biofilms on microtiter plates or pegs were carefully rinsed three times with 1× PBS to discard planktonic cells and exposed at 65 °C until completely dry. Biofilms were then fully covered with a 1% CV stain solution for 10 min at room temperature [52]. The CV was then removed, washed with 1× PBS to eliminate the excess of dye and heat-dried for 60 min. Biofilm formation was quantified by eluting the CV fixed to the biofilm in 33% glacial acetic acid and absorbance of each well was measured at 580 nm using a microplate spectrophotometer (EPOCH, BioTek, Winooski, VT, USA).

The minimum biofilm inhibitory concentration (MBIC) was defined as the minimal concentration of the compound that led to a three-fold decrease in absorbance when compared to the growth control values. All the experiments were carried out in triplicate.

### 4.7. Antimicrobial Synergy Study

Two-dimensional checkerboard arrays were used to determine the efficacy of the combination of the selected gold(III) complex, **2b**, together with colistin (CST), in contrast to their individual activities. This experiment was carried out only with the CST-resistant strain *E. coli* GN1044, with the aim of observing differences in their resistance profile. Gram positive strains were not included since they are intrinsically resistant to polymyxins.

The setup of each assay plate evaluated two-fold dilutions of selected gold(III) with two-fold dilutions of CST, considering a concentration range that included, in the middle of the gradient, the MIC of each molecule against the tested strain. Inoculum size, culture media and incubation conditions were the same as described above for the MIC microdilution method. Positive and negative controls were also included, and the final volume of the assay was 200 μL per well. The fractional inhibitory concentration index (FICI) was calculated considering the following formula: FICI = FIC A + FIC B, where FIC A is the MIC of drug A in combination/MIC of drug A alone, and FIC B is the MIC of drug B in combination/MIC of drug B alone. Synergy was defined by a FICI ≤ 0.5. Interactions with FICI values ranging from 0.5 to 4 were classified as additive/indifferent. Antagonism was defined by a FICI ≥ 4 [55].

### 4.8. In Vitro Cytotoxicity Assay

The cytotoxicity of gold(III) complexes was determined using a colorimetric kit that detects cellular metabolic activities (XTT Cell Proliferation Assay Kit, Canvax Biotech, Córdoba, Spain). The experiment is based on the extracellular reduction of XTT (sodium 2,3-bis(2-methoxy-4-nitro-5-sulfophenyl)-5-[(phenylamino)carbonyl]-2*H*-tetrazolium) by NADH to a highly colored formazan dye. This only occurs in metabolically active cells as it depends on mitochondrial respiration. Therefore, the amount of formazan produced is proportional to the viable cells in the sample. The experiment was also carried out with DMSO as a control, to see if the solvent caused toxicity. We used Jurkat E6.1 commercial cell line. The cells were maintained in RPMI-1470 supplemented with 10% fetal bovine serum (FBS), penicillin and streptomycin at 37 °C and 5% CO_2_ atmosphere.

Study was conducted in sterile 96-well microplates and cells were spread to a density of 10^5^ in 100 μL/well. Serial concentrations of the tested compounds were also included in the rows, starting with a range of 64 mg/L. After 24 h incubation with the conditions mentioned before, we added 50 μL of XTT to each well and incubated for an additional 4 h. Measurement of the results were made on the Epoch™ spectrophotometer plate reader at a wavelength of 450–500 nm and 630–690 nm. The first measurement gives the signal absorbance of the samples, and the second gives the background signal.

### 4.9. Statistical Analysis

Data analysis was performed with the statistical software R commander 4.1.0. Regarding the assessment of biofilm formation, determination of the appropriate protocol was carried out using the analysis of variance (ANOVA) test in order to check for significant differences between the conditions. However, our data did not comply with the homoscedasticity assumption (i.e., no equal variances), so heteroscedastic ANOVA were used. This test employs Welch’s correction and allows comparison of normal samples with different variances. After establishing differences between means, we used the Bonferroni posthoc test for pairwise comparisons. Graphics were performed using GraphPad Prism 9 software.

## 5. Conclusions

In conclusion, gold(III) complexes have demonstrated their potential antimicrobial activity both in monotherapy against MDR bacteria and in combination with colistin against Gram-negative colistin-resistant bacteria. This association enhances the activity of both drugs and significantly reduces their therapeutic concentrations, which is particularly interesting in the case of colistin, which is a drug widely used in the clinical and veterinary setting, to avoid the emergence of resistant mutants and reduce its known toxicity. Although the mechanism appears to be membrane-related, further studies are needed to fully decipher its molecular targets. Given their antibacterial and antibiofilm activity, considering the high resistance profile of the strains tested here, and their promising in vitro toxicity, they appear to be good candidates to become a new antibacterial agent.

## 6. Patents

A gold(III) complex, a conjugate of the gold(III) complex, a pharmaceutical composition comprising the gold(III) complex and uses and a process for preparing the gold(III) complex. WO 2019211222 A1 (7 November 2019).

## Figures and Tables

**Table 1:** Summary of the MIC values (mg/L) of the (C^S)-cyclometallated Au(III) complexes and standard drugs under investigation.

Species	Reference	1	2a	2b	2c	2d	2e	2f	3	CIP	CST	AMP	SXT	VAN
*Salmonella rissen*	E3G2V1C7R	64	16	8	8	16	32	8	8	0.25	0.25	128	4	-
*Salmonella panama*	E3G3V2C1	64	16	8	8	16	64	8	16	0.1	0.5	128	8	-
*Salmonella anatum*	E3G2V1C34R	64	16	8	8	16	64	8	16	0.25	0.25	128	16	-
*E. coli*	GN1044	64	16	8	8	16	32	8	8	0.015	32	128	16	-
*E. coli*	GN1035	64	16	8	8	16	64	4	8	0.5	8	128	16	-
*E. coli*	2462 ME2-1	64	16	16	8	16	32	8	8	1	0.125	128	16	-
*E. coli*	2461 ME2-2	64	16	8	8	16	32	8	8	1	0.125	128	16	-
*E. coli*	GN444	64	16	16	8	16	32	4	16	1	0.125	128	16	-
*E. coli*	173	64	16	16	16	16	32	8	8	1	0.125	128	16	-
*Pseudomonas* *aeruginosa*	3162PE1	64	32	16	16	32	64	8	16	0.125	0.5	64	-	-
*Staphylococcus* *haemolyticus*	620DD1	16	0.03	0.5	1	0.125	0.5	0.25	0.125	-	-	1	-	2
*Staphylococcus* *xylosus*	930DE1	16	0.25	0.5	1	0.25	1	0.5	0.25	-	-	0.125	-	4
*Staphylococcus* *chromogenes*	401-PD1	8	0.25	0.125	0.5	0.125	0.25	0.25	0.06	-	-	2	-	2
*Staphylococcus* *chromogenes*	21AD-1	8	0.125	0.125	0.5	0.06	0.5	0.5	0.125	-	-	2	-	2
*Staphylococcus* *chromogenes*	7439PE-1	8	0.06	0.25	0.125	0.06	0.5	0.125	0.06	-	-	8	-	2
*Streptococcus uberis*	380 ME-3	32	1	1	2	2	8	1	0.5	-	-	64	-	1

CIP, ciprofloxacin; CST, colistin; AMP, ampicillin; SXT, trimethoprim-sulphametoxazole; VAN, vancomycin. Values are the mean of three replicates.

**Table 2:** Summary of the MBC values (mg/L) of the (C^S)-cyclometallated Au(III) complexes and standard drugs under investigation.

Species	Reference	1	2a	2b	2c	2d	2e	2f	3	CST	VAN
*Salmonella rissen*	E3G2V1C7R	64	64	16	16	16	64	32	128	0.25	-
*Salmonella panama*	E3G3V2C1	128	64	32	32	16	64	16	64	0.5	-
*Salmonella anatum*	E3G2V1C34R	64	64	32	32	16	64	16	64	0.25	-
*E. coli*	GN1044	64	32	16	16	16	64	16	64	16	-
*E. coli*	GN1035	64	64	32	32	32	64	32	64	16	-
*E. coli*	2462 ME2-1	64	64	16	16	16	64	32	64	0.25	-
*E. coli*	2461 ME2-2	64	16	8	8	16	64	16	32	0.125	-
*E. coli*	GN444	64	16	16	8	16	64	8	32	0.25	-
*E. coli*	173	64	16	16	16	16	64	32	32	0.125	-
*Pseudomonas aeruginosa*	3162PE1	128	32	32	32	64	64	32	32	1	-
*Staphylococcus haemolyticus*	620DD1	16	0.125	0.5	1	0.25	1	0.5	0.25	-	2
*Staphylococcus xylosus*	930DE1	16	0.5	0.5	1	0.5	1	2	0.5	-	4
*Staphylococcus chromogenes*	401-PD1	16	0.5	0.125	1	0.125	1	0.5	0.125	-	2
*Staphylococcus chromogenes*	21AD-1	16	0.25	0.125	2	0.25	0.5	0.5	0.125	-	2
*Staphylococcus chromogenes*	7439PE-1	8	0.06	0.25	0.25	0.25	0.5	0.5	0.06	-	2
*Streptococcus uberis*	380 ME-3	128	16	81	8	16	16	8	16	-	2

CST, colistin; VAN, vancomycin. Values are the mean of three replicates.

**Table 3:** Summary of the MBIC values (mg/L) of the (C^S)-cyclometallated Au(III) complexes and standard antibiotics under investigation.

Species	Reference	1	2a	2b	2c	2d	2e	2f	3	CST	VAN
*Salmonella panama*	E3G3V2C1	16	64	8	8	8	>64	8	16	4	-
*Salmonella anatum*	E3G2V1C34R	16	>64	16	16	16	>64	32	32	4	-
*E. coli*	2462 ME2-1	16	32	8	4	16	>64	4	16	2	-
*E. coli*	2461 ME2-2	16	32	8	4	16	>64	4	8	2	-
*E. coli*	GN444	16	32	8	8	16	>64	4	8	4	-
*Staphylococcus haemolyticus*	620DD1	>32	>32	>32	>32	>32	>32	>32	>32	-	16
*Staphylococcus chromogenes*	401-PD1	>32	>32	>32	>32	>32	>32	>32	>32	-	16
*Staphylococcus chromogenes*	21AD-1	>32	>32	>32	>32	>32	>32	>32	>32	-	16

CST, colistin; VAN, vancomycin.

## Data Availability

The data presented in this article is contained within the article and the Supplementary materials.

## Supplementary Materials

The following are available online at https://www.mdpi.com/article/10.3390/antibiotics11121728/s1, Synthesis of Au(III) complexes; NMR spectra of Au(III) complexes; Figure S1. ^1^H NMR spectrum of **2a** in CDCl_3_; Figure S2. ^13^C NMR spectrum of **2a** in CDCl_3_; Figure S3. ^31^P NMR spectrum of **2a** in CDCl_3_; [Au(dppta)(morpholinyldithiocarbamate)] [PF_6_] **2d**; Figure S4. ^1^H NMR spectrum of **2d** in acetone-d_6_; Figure S5. ^13^C NMR spectrum of **2d** in acetone-d_6_; Figure S6. ^31^P NMR spectrum of **2d** in acetone-d_6_; [Au(dppta)(piperazinyldithiocarbamate)] [PF_6_] **2e**; Figure S7. ^1^H NMR spectrum of **2e** in CD_3_CN; Figure S8. ^13^C NMR spectrum of **2e** in CD_3_CN; Figure S9. ^31^P NMR spectrum of **2e** in CD_3_CN [Au(dppta)(azepanyldithiocarbamate)] [PF_6_] **2f**; Figure S10. ^1^H NMR of Au(III) complex **2f** in CDCl_3_; Figure S11. ^13^C NMR of Au(III) complex **2f** in CDCl_3_; Figure S12. ^31^P NMR of Au(III) complex **2f** in CDCl_3_; [Au(bppta)(diethyldithiocarbamate)] [PF_6_] **3**; Figure S13. ^1^H NMR of Au(III) complex **3** in CDCl_3_; Figure S14. ^13^C NMR of Au(III) complex **3** in CDCl_3_; Figure S15. ^31^P NMR of Au(III) complex **3** in CDCl_3_.
