# Supplementary material for: Gold(III) Complexes Activity against Multidrug-Resistant Bacteria of Veterinary Significance
Source: Antibiotics (Basel). 2022 Dec 1;11(12):1728. doi: 10.3390/antibiotics11121728 (PMC9774617; doi:10.3390/antibiotics11121728)
Supplement: Supplementary file 1 [file antibiotics-11-01728-s001.zip › antibiotics-2052263-supplementary.pdf]

Supporting Information

Gold(III) Complexes Activity Against Multidrug-Resistant Bacteria Of Clinical And Veterinary Significance

Carlos Ratia[1], Sara Sueiro[1], Raquel G. Soengas[2], María José Iglesias[3], Fernando López Ortiz[3*], Sara M. Soto[1,4*]

[1]ISGlobal, Hospital Clínic—Universitat de Barcelona, 08036 Barcelona, Spain.

[2]Departamento de Química Orgánica e Inorgánica, Universidad de Oviedo, Julián Clavería 7, 33006 Oviedo, SPAIN.

[3]Área de Química Orgánica, Centro de Investigación CIAIMBITAL, Universidad de Almería, 04120 Almería, Spain.

[3]CIBER Enfermedades Infecciosas (CIBERINFEC), Instituto de Salud Carlos III, 28029 Madrid, Spain.

Contents

1. Synthesis of Au(III) complexes....S3

1.1. General materials and methods....S3

1.2. Method M1: Synthesis of [Au(dppta)(dtc)][PF_6] complexes **2a-f**....S3

1.3. Method M2: Synthesis of [Au(bppta)(dtc)][PF_6] complex **3**....S4

2. References....S5

3. NMR spectra of Au(III) complexes....S6

[Au(dppta)(dimethyldithiocarbamate)] [PF_6] **2a**....S6

Figure S1. ^{1}H NMR spectrum of **2a** in $CDCl_3$.

Figure S2. ^{13}C NMR spectrum of **2a** in $CDCl_3$.

Figure S3. ^{31}P NMR spectrum of **2a** in $CDCl_3$.

[Au(dppta)(morpholinyldithiocarbamate)] [PF_6] **2d**....S8

Figure S4. ^{1}H NMR spectrum of **2d** in acetone-d_6.

Figure S5. ^{13}C NMR spectrum of **2d** in acetone-d_6.

Figure S6. ^{31}P NMR spectrum of **2d** in acetone-d_6.

[Au(dppta)(piperazinyldithiocarbamate)] [PF_6] **2e**....S10

Figure S7. ^{1}H NMR spectrum of **2e** in CD_3CN.

Figure S7. ^{13}C NMR spectrum of **2e** in CD_3CN.

Figure S9. ^{31}P NMR spectrum of **2e** in CD_3CN.

[Au(dppta)(azepanyldithiocarbamate)] [PF_6] **2f**..........S12

Figure S10. ^{1}H NMR of Au(III) complex **2f** in $CDCl_3$.

Figure S11. ^{13}C NMR of Au(III) complex **2f** in $CDCl_3$.

Figure S12. ^{31}P NMR of Au(III) complex **2f** in $CDCl_3$.

[Au(bppta)(diethyldithiocarbamate)] [PF_6] **3**..........S14

Figure S13. ^{1}H NMR of Au(III) complex **3** in $CDCl_3$.

Figure S14. ^{13}C NMR of Au(III) complex **3** in $CDCl_3$.

Figure S15. ^{31}P NMR of Au(III) complex **3** in $CDCl_3$.

1. Synthesis of Au(III) complexes

1.1 General materials and methods. All reactions were carried out under inert atmosphere, in previously dried Schlenks. All new compounds were characterized based on their NMR spectroscopy data and high resolution mass spectra. NMR spectra were obtained on a Bruker Avance III HD 300 (^{1}H 300.13 MHz; ^{13}C 75.47 MHz; ^{31}P 121.49 MHz), Bruker Avance III HD 500 (^{1}H 500.13 MHz; ^{13}C 125.76 MHz; ^{31}P 202.46 MHz) and Bruker Avance III HD 600 (^{1}H 600.13 MHz; ^{13}C 150.91 MHz). Chemical shifts are given in ppm using tetramethylsilane (TMS) for ^{1}H and ^{13}C as internal standards and 85% H_3PO_4 for ^{31}P as external standard. The following abbreviations are used to indicate the multiplicity of signal: s – singlet, d – doublet, t – triplet, q – quartet and sep – septet. High Resolution Mass Spectra (HRMS) were recorded on an Agilent Technologies LC/MSD-TOF and HP 1100 MSD spectrometer using electrospray ionization. Melting points were recorded on Büchi B-540 capillary melting point apparatus and are uncorrected.

1.2. Method M1: Synthesis of [Au(dppta)(dtc)][PF$_6$] complexes 2a-f

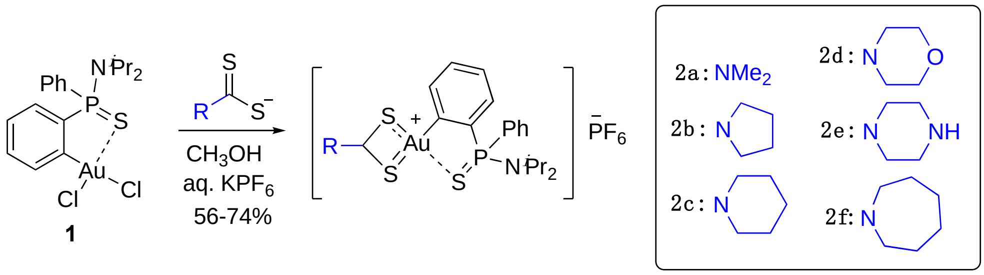

To a solution of [Au(dppta)Cl$_2$] complex **1** (117 mg, 0.20 mmol) in MeOH (6 mL), the corresponding dithiocarbamate salt was added (0.20 mmol). The reaction mixture was stirred at rt for 12 h and then aqueous saturated potassium hexafluorophosphate was added and the mixture was stirred for 15 min. After partial evaporation of the methanol, the resulting solid was filtered and the residue was washed with water and diethyl ether to afford desired [Au(dppta)(dtc)][PF$_6$] complexes **2a-f** (56-74% yield). The spectroscopic data obtained for the known complexes **2b** and **2c** agree with those described in the literature (Soto et al, 2019). The complete characterization of the new complexes **2a**, **2d-f** is shown below.

[Gold (III) 2-((diisopropylamino)(phenyl)phosphorothioyl)phenyl) (dimethyldithiocarbamate)] hexafluorophosphate (2a): Yellow solid (115 mg, 74%). HRMS (ESI^{+}) [M]$^{+}$ calcd. for $C_{21}H_{29}AuN_2PS_3$, 633.0889; found, 633.0891. IR (KBr, ν cm^{-1}): 556 (P=S), 840 (PF$_6$), 1576 (NCS$_2$). ^{1}H NMR (300 MHz, CDCl$_3$) δ 1.21 (d, $^{3}J_{HH}$ 6.8 Hz, 6H, H12), 1.30 (d, $^{3}J_{HH}$ 6.8 Hz, 6H, H12), 3.42 (s, 3H, CH$_3$), 3.43 (s, 3H, CH$_3$), 3.71 (dh, $^{3}J_{PH}$ 20.4 Hz, $^{3}J_{HH}$ 6.8 Hz, 2H, H11), 7.37 (ddd, $^{3}J_{HH}$ 7.7, $^{3}J_{PH}$ 3.6, $^{4}J_{HH}$ 1.2 Hz, 1H, H6), 7.49 (tt, $^{3}J_{HH}$ 7.4, $^{4}J_{HH}$ 1.8, $^{4}J_{PH}$ 1.8 Hz, 1H, H5), 7.57 (ddd, J = $^{3}J_{HH}$ 7.5, $^{3}J_{PH}$ 4.4, $^{4}J_{HH}$ 1.2 Hz, 1H, H9), 7.59-7.71 (m, 3H, H4, H9, H10), 7.75 (dt, $^{3}J_{HH}$ 7.6, $^{4}J_{HH}$ 1.6, $^{4}J_{PH}$ 1.6 Hz, 1H, H3), 8.11 (ddd, $^{3}J_{PH}$ 14.4, $^{3}J_{HH}$ 8.3, $^{4}J_{HH}$ 1.4 Hz, 2H, H8). ^{13}C NMR (75 MHz, CDCl$_3$) δ 23.1 (d, $^{3}J_{PC}$ 3.0 Hz, C12), 23.4 (d, $^{3}J_{PC}$ 3.4 Hz, C12), 40.6 (CH$_3$), 40.8 (CH$_3$), 50.8 (d, $^{2}J_{PC}$ 3.2 Hz, C11), 127.5 (d, $^{1}J_{PC}$ 101.6 Hz, C7), 128.5 (d, $^{2}J_{PC}$ 12.0 Hz, C6), 129.7 (d, $^{3}J_{PC}$ 13.7 Hz, C9), 132.6 (d, $^{3}J_{PC}$ 16.9 Hz, C5), 133.1 (d, $^{3}J_{PC}$ 12.3 Hz, C3), 133.5 (d, $^{2}J_{PC}$ 11.8 Hz, C8), 134.8 (d, $^{4}J_{PC}$ 3.2 Hz, C10), 135.4 (d, $^{4}J_{PC}$ 3.6 Hz, C4), 138.7 (d, $^{1}J_{PC}$ 124.0 Hz, C1), 143.7 (d, $^{2}J_{PC}$ 27.6 Hz, C2), 196.1 (NCS$_2$) ppm. ^{31}P NMR (121 MHz, CDCl$_3$) δ -143.6 (sep, $^{1}J_{PF}$ 712.3 Hz), 76.4 ppm.

[Gold(III) 2-((diisopropylamino)(phenyl)phosphorothioyl)phenyl) (morpholin-1-dithiocarbamate)] hexafluorophosphate (2d): Yellow solid (104 mg, 63%). HRMS (ESI^+) $[M]^+$ calcd. for $C_{23}H_{3i}AuON_2PS_3$, 675.1002; found, 675.1017. IR (KBr, ν cm^{-1}): 557 (P=S), 840 (PF_6), 1548 (NCS_2). ^1H NMR (300 MHz, acetone-d_6) δ 1.30 (d, $^3J_{HH}$ 6.8 Hz, 6H, H12), 1.39 (d, $^3J_{HH}$ 6.8 Hz, 6H, H12), 3.81-4.04 (m, 6H, H11, 2 x CH_2), 4.10-4.12 (m, 4H, 2 x CH_2), 7.49-7.56 (m, 1H, H5), 7.66-7.73 (m, 2H, H9), 7.74-7.82 (m, 2H, H6, H10), 7.84-7.91 (m, 1H, H3), 8.10-8.22 (m, 2H, H8) ppm. ^{13}C NMR (75 MHz, acetone-d_6) δ 22.4 (d, $^3J_{PC}$ 3.0 Hz, C12), 22.6 (d, $^3J_{PC}$ 3.5 Hz, C12), 49.6 (2 x CH_2), 50.7 (d, $^2J_{PC}$ 3.2 Hz, C11), 65.3 (2 x CH_2), 127.9 (d, $^1J_{PC}$ 101.7 Hz, C7), 128.5 (d, $^3J_{PC}$ 12.2 Hz, C6), 129.6 (d, $^3J_{PC}$ 13.7 Hz, C9), 132.5 (d, $^3J_{PC}$ 16.8 Hz, C5), 133.7 (d, $^3J_{PC}$ 12.0 Hz, C3), 134.8 (d, $^4J_{PC}$ 3.2 Hz, C10), 135.6 (d, $^4J_{PC}$ 3.5 Hz, C4), 138.8 (d, $^1J_{PC}$ 124.1 Hz, C1), 143.8 (d, $^2J_{PC}$ 27.6 Hz, C2), 194.4 (NCS_2) ppm. ^{31}P NMR (121 MHz, acetone-d_6) δ -143.5 (sep, $^1J_{PF}$ 712.5 Hz), 75.5 ppm.

[Gold(III) 2-((diisopropylamino)(phenyl)phosphorothioyl)phenyl) (piperazin-1-dithiocarbamate)] hexafluorophosphate (2e): Yellow solid (92 mg, 56%). Recrystallized from acetonitrile. M.p. 203 ºC. HRMS (ESI^+) $[M]^+$ calcd. for $C_{23}H_{32}AuN_3PS_3$, 674.1162; found, 674.1153. IR (KBr, ν cm^{-1}): 557 (P=S), 840 (PF_6), 1532 (NCS_2). ^1H NMR (300 MHz, CD_3CN) δ 1.20 (d, $^3J_{HH}$ 6.8 Hz, 6H, H12), 1.30 (d, $^3J_{HH}$ 6.8 Hz, 6H, H12), 3.43-4.45 (m, 4H, 2 x CH_2), 3.79 (dh, $^3J_{PH}$ 18.4 Hz, $^3J_{HH}$ 6.8 Hz, 2H, H11), 4.16-4.24 (m, 4H, 2 x CH_2), 7.39-7.43 (m, 1H, H5), 7.54-7.62 (m, 2H, H6, H10), 7.64-7.71 (m, 2H, H9), 7.75-7.81 (m, 1H, H4), 7.84-7.91 (m, 1H, H3), 8.14-8.22 (m, 2H, H8) ppm. ^{13}C NMR (75 MHz, CD_3CN) δ 22.3 (d, $^3J_{PC}$ 3.0 Hz, C12), 22.5 (d, $^3J_{PC}$ 3.3 Hz, C12), 42.1 (CH_3), 45.3 (CH_3), 50.7 (d, $^2J_{PC}$ 3.3 Hz, C11), 127.6 (d, $^1J_{PC}$ 101.8 Hz, C7), 128.5 (d, $^2J_{PC}$ 12.3 Hz, C6), 129.6 (d, $^3J_{PC}$ 13.8 Hz, C9), 132.5 (d, $^3J_{PC}$ 16.8 Hz, C5), 133.7 (d, $^3J_{PC}$ 12.0 Hz, C3), 133.7 (d, $^2J_{PC}$ 11.9 Hz, C8), 134.8 (d, $^4J_{PC}$ 2.9 Hz, C4), 135.7 (d, $^4J_{PC}$ 3.4 Hz, C10), 138.6 (d, $^1J_{PC}$ 124.1 Hz, C1), 143.2 (d, $^2J_{PC}$ 27.5 Hz, C2), 198.4 ppm. ^{31}P NMR (121 MHz, CD_3CN) δ -143.9 (sep, $^1J_{PF}$ 707.0 Hz), 74.9 ppm.

[Gold(III) 2-((diisopropylamino)(phenyl)phosphorothioyl)phenyl) (azepan-1-dithiocarbamate)] hexafluorophosphate (2f): Yellow solid (99 mg, 60%). Recrystallized from acetonitrile. M.p. 126 ºC. HRMS (ESI^+) $[M]^+$ calcd. for $C_{25}H_{35}AuN_2PS_3$, 687.1355; found, 687.1360. IR (KBr, ν cm^{-1}): 557 (P=S), 839 (PF_6), 1540 (NCS_2). ^1H NMR (300 MHz, $CDCl_3$) δ 1.19 (d, $^3J_{HH}$ 6.8 Hz, 6H, H12), 1.29 (d, $^3J_{HH}$ 6.8 Hz, 6H, H12), 1.50-1.70 (m, 4H, 2 x CH_2), 1.78-1.98 (m, 4H, 2 x CH_2), 3.71 (dh, $^2J_{HH}$ 17.9 Hz, $^3J_{HH}$ 6.8 Hz, 2H, H11), 3.82-3.92 (m, 4H, 2 x CH_2), 7.37 (ddd, $^3J_{HH}$ 7.7 Hz, $^3J_{PH}$ 3.5 Hz, $^4J_{HH}$ 1.7 Hz, 1H, H5), 7.46 (tt, $^3J_{HH}$ 7.5 Hz, $^3J_{PH}$ 7.5 Hz, $^4J_{HH}$ 1.7 Hz, 1H, H10), 7.55-7.67 (m, 3H, H6, H9), 7.69-7.74 (m, 1H, H4), 7.74-7.80 (m, 1H, H3), 8.04-8.17 (m, 2H, H8) ppm. ^{13}C NMR (75 MHz, $CDCl_3$) δ 23.0 (d, $^3J_{PC}$ 3.1 Hz, C12), 23.3 (d, $^3J_{PC}$ 3.4 Hz, C12), 26.3 (CH_2), 26.5 (CH_2), 26.58 (CH_2), 26.61 (CH_2), 50.7 (d, $^2J_{PC}$ 3.3 Hz, C11), 52.1 (CH_2), 52.4 (CH_2), 127.4 (d, $^1J_{PC}$ 101.6 Hz, C7), 128.4 (d, $^2J_{PC}$ 12.0 Hz, C6), 129.6 (d, $^3J_{PC}$ 13.7 Hz, C9), 132.5 (d, $^3J_{PC}$ 17.0 Hz, C5), 133.1 (d, $^3J_{PC}$ 12.4 Hz, C3), 133.4 (d, $^2J_{PC}$ 11.7 Hz, C8), 134.7 (d, $^4J_{PC}$ 3.2 Hz, C4), 135.3 (d, $^4J_{PC}$ 3.6 Hz, C10), 138.6 (d, $^1J_{PC}$ = 124.2 Hz, C1), 143.9 (d, $^2J_{PC}$ 28.8 Hz, C2), 195.1 (NCS_2) ppm. ^{31}P NMR (121 MHz, $CDCl_3$) δ -143.6 (sep, $^1J_{PF}$ 712.5 Hz), 76.5 ppm.

1.3. Method M2: Synthesis of [Au(bppta)(dtc)][PF_6] complex 3

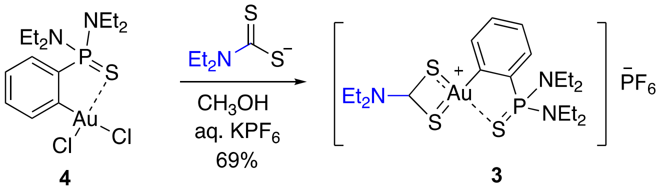

4 **3**

To a solution of [Au(bppta)Cl_2] complex **4** (110 mg, 0.20 mmol) in MeOH (6 mL), sodium diethyl dithiocarbamate was added (34 mg, 0.20 mmol). The reaction mixture was stirred at rt for 12 h and then aqueous saturated potassium hexafluorophosphate was added and the mixture was stirred for 15 min. After partial evaporation of the methanol, the resulting solid was filtered and the residue was washed with water and diethyl ether to afford [Au(bppta)(Et_2NCS_2)][PF_6] complex **3** (111 mg, 69%) as a yellow solid.

Gold (III) 2-(bis(diethylamino)(phosphorothioyl)phenyl) (diethyldithiocarbamate)] hexafluorophosphate (3): HRMS (ESI^+) $[M]^+$ calcd. for $C_{19}H_{33}AuN_2PS_3$, 620.1313; found, 628.1323. IR (KBr, ν cm^{-1}): 557 (P=S), 838 (PF_6), 1552 (NCS_2). ^1H NMR (300 MHz, $CDCl_3$) δ 1.17 (t, $^3J_{HH}$ 7.1 Hz, 12H, C8), 1.43 (t, $^3J_{HH}$ 7.2 Hz, 6H, 2 x CH_3), 3.27 (dq, $^3J_{PH}$ 14.0, $^3J_{HH}$ 7.0 Hz, 8H, H7), 3.85 (q, $^3J_{HH}$ 7.4 Hz, 4H, 2 x CH_2), 7.48 (m, 4H, H3, H4, H5, H6) ppm. ^{13}C NMR (75 MHz, $CDCl_3$) δ 12.3 (CH_3), 12.5 (CH_3), 13.6 (CH_3), 13.7 (CH_3), 40.6 (CH_2), 40.7 (CH_2), 47.0 (d, $^3J_{PC}$ 12.8 Hz, C7), 128.3 (d, $^3J_{PC}$ 12.8 Hz, C5), 131.7 (d, $^3J_{PC}$ 12.7 Hz, C3), 132.2 (d, $^2J_{PC}$ 17.7 Hz, C6), 135.2 (d, $^4J_{PC}$ 3.6 Hz, C4), 136.3 (d, $^1J_{PC}$ 143.0 Hz, C1), 142.7 (d, $^2J_{PC}$ 28.1 Hz, C2), 195.2 (NCS_2) ppm. ^{31}P NMR (121 MHz, $CDCl_3$) δ -143.6 (sep, $^1J_{PF}$ 712.3 Hz), 76.4 ppm.

2. References

Soto, S., Ratia C., Cepas, V., López, Y., López-Ortiz, F.; Iglesias, M. J., Soengas, R. G. (2019). A gold(III) complex, a conjugate of the gold(III) complex, a pharmaceutical composition comprising the gold(III) complex and uses and a process for preparing the gold(III) complex. WO 2019211222 A1, 20191107.

3. NMR spectra of Au(III) complexes

[Gold (III) 2-((diisopropylamino)(phenyl)phosphorothioyl)phenyl) (dimethyldithiocarbamate)] hexafluorophosphate (2a)

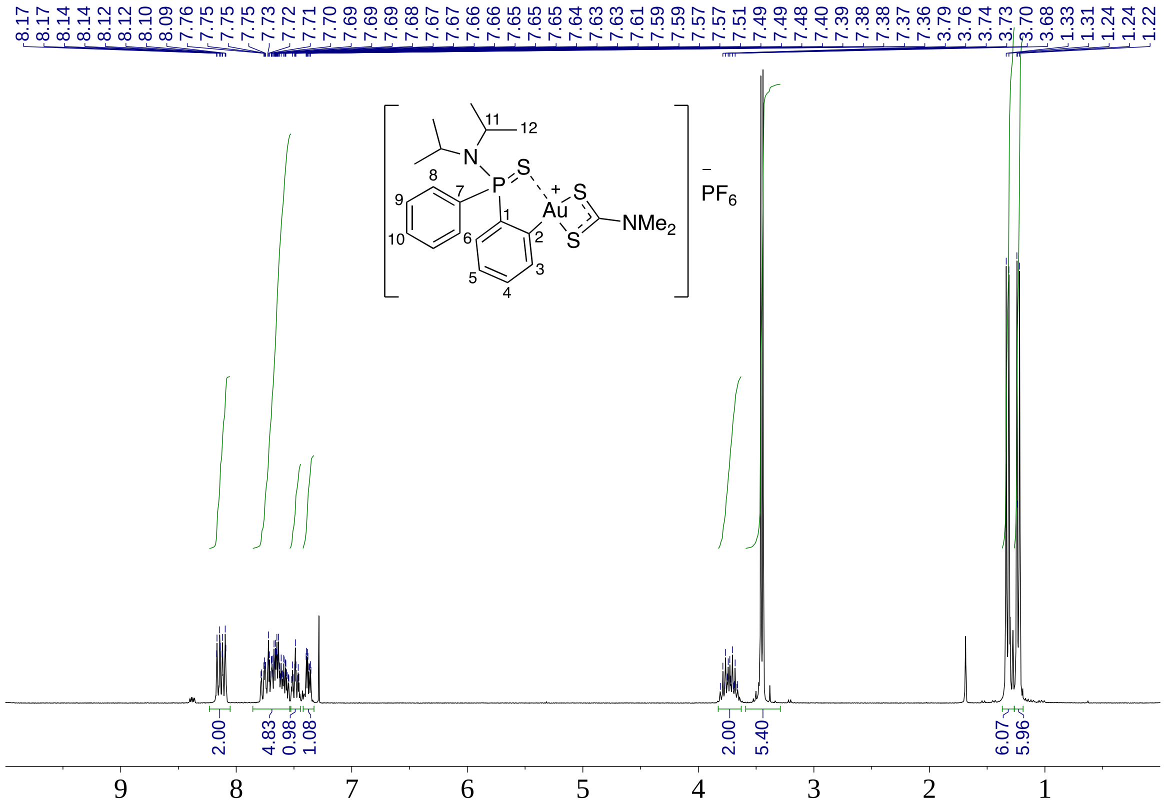

Figure S1. ^1H NMR spectrum of **2a** in $CDCl_3$.

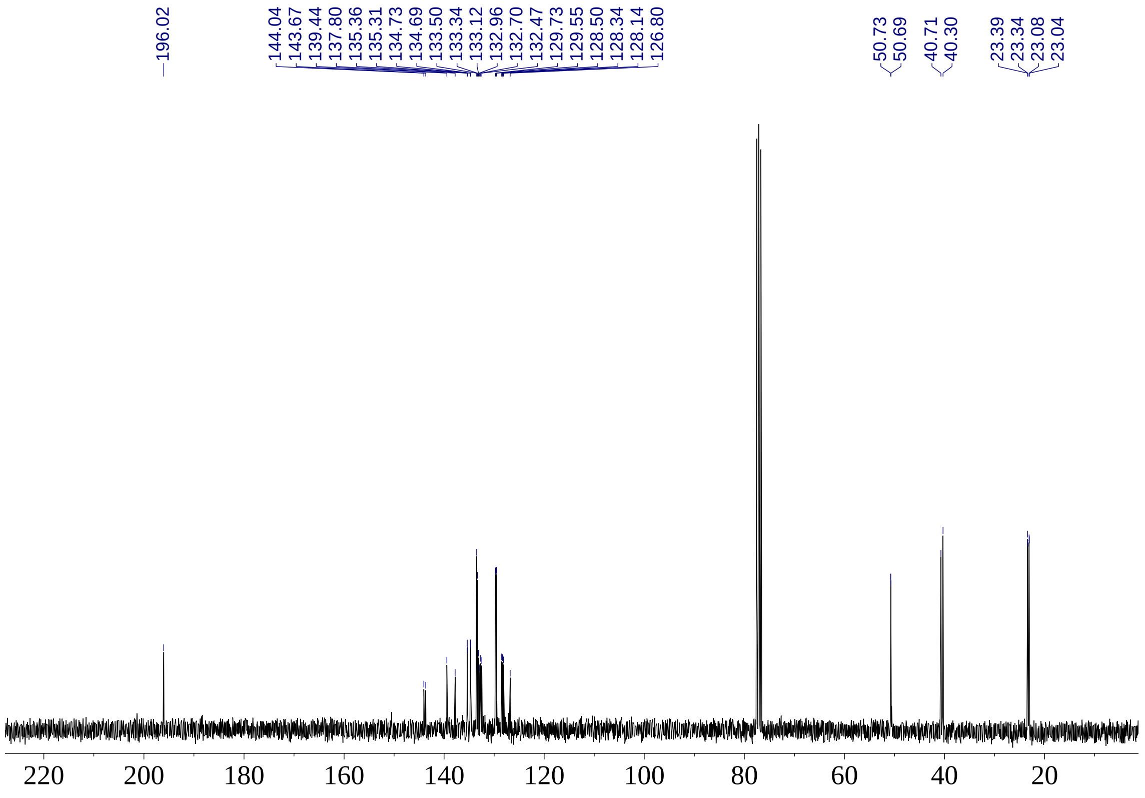

Figure S2. ^{13}C NMR spectrum of **2a** in $CDCl_3$.

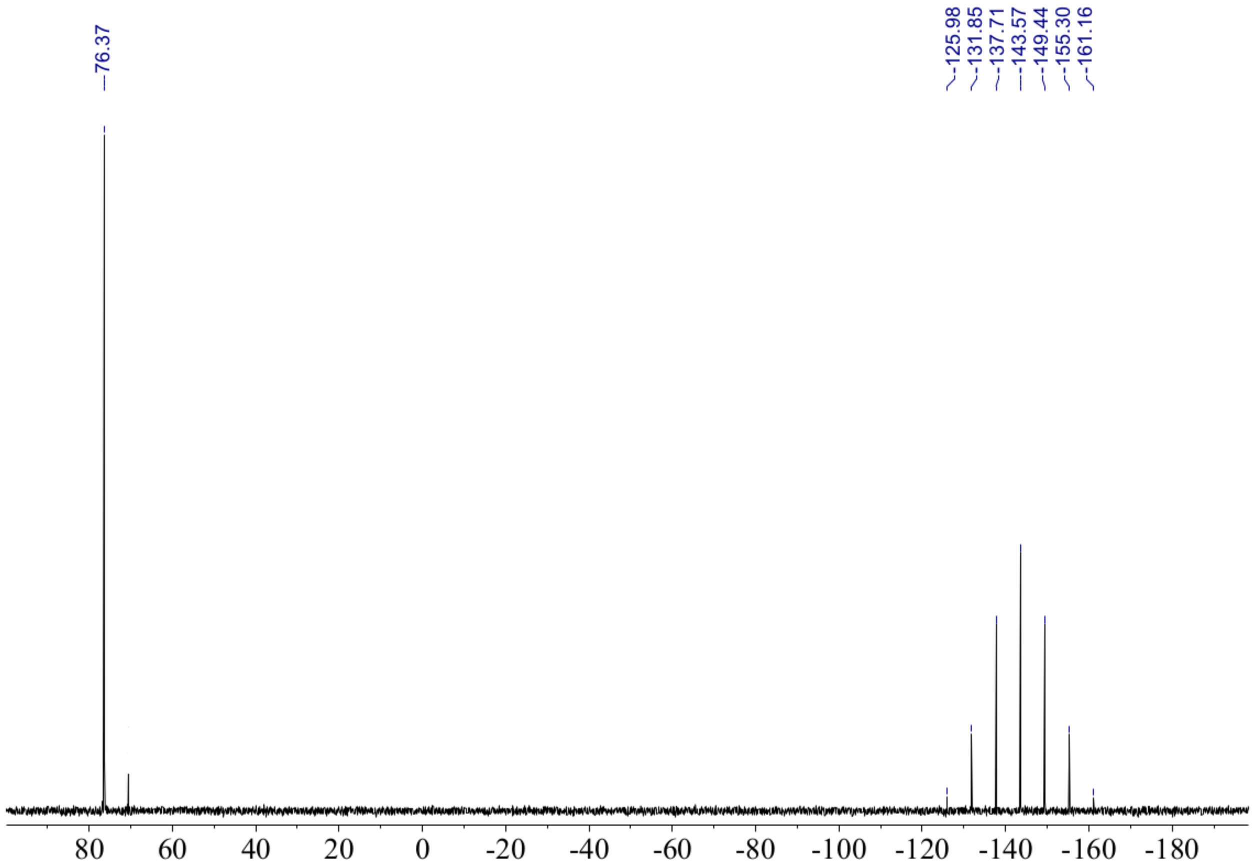

Figure S3. ^{31}P NMR spectrum of **2a** in $CDCl_3$.

[Gold(III) 2-((diisopropylamino)(phenyl)phosphorothioyl)phenyl) (morpholin-1-dithiocarbamate)] hexafluorophosphate (2d)

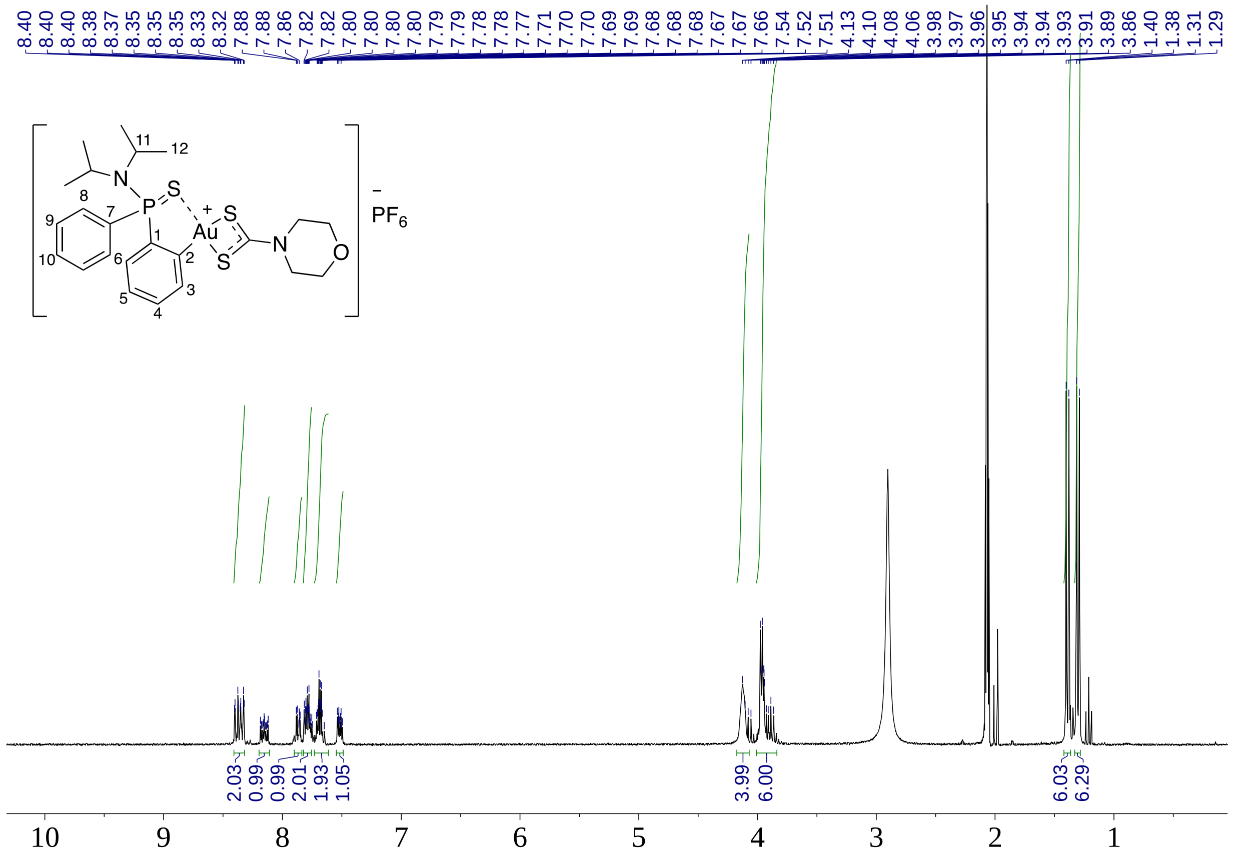

Figure S4. ^{1}H NMR spectrum of **2d** in acetone-d$_6$.

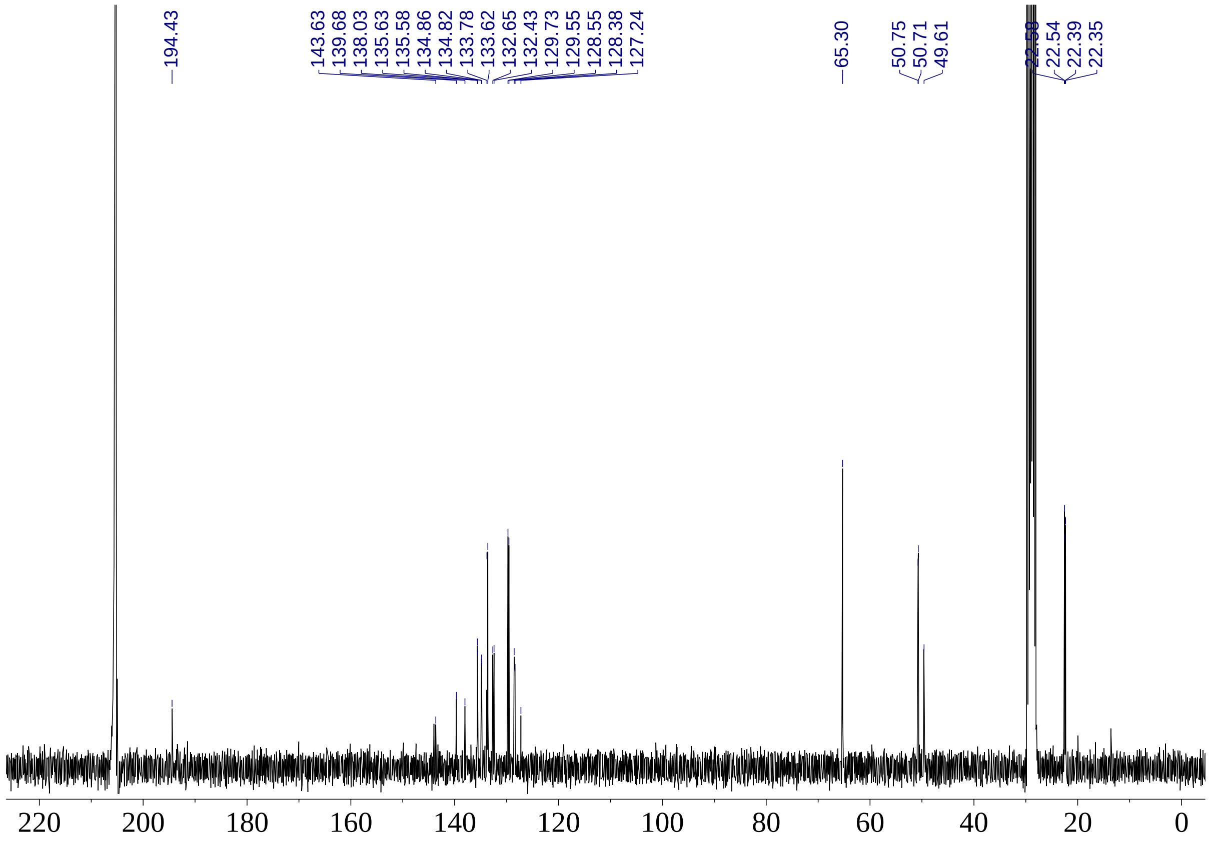

Figure S5. ^{13}C NMR spectrum of **2d** in acetone-d$_6$.

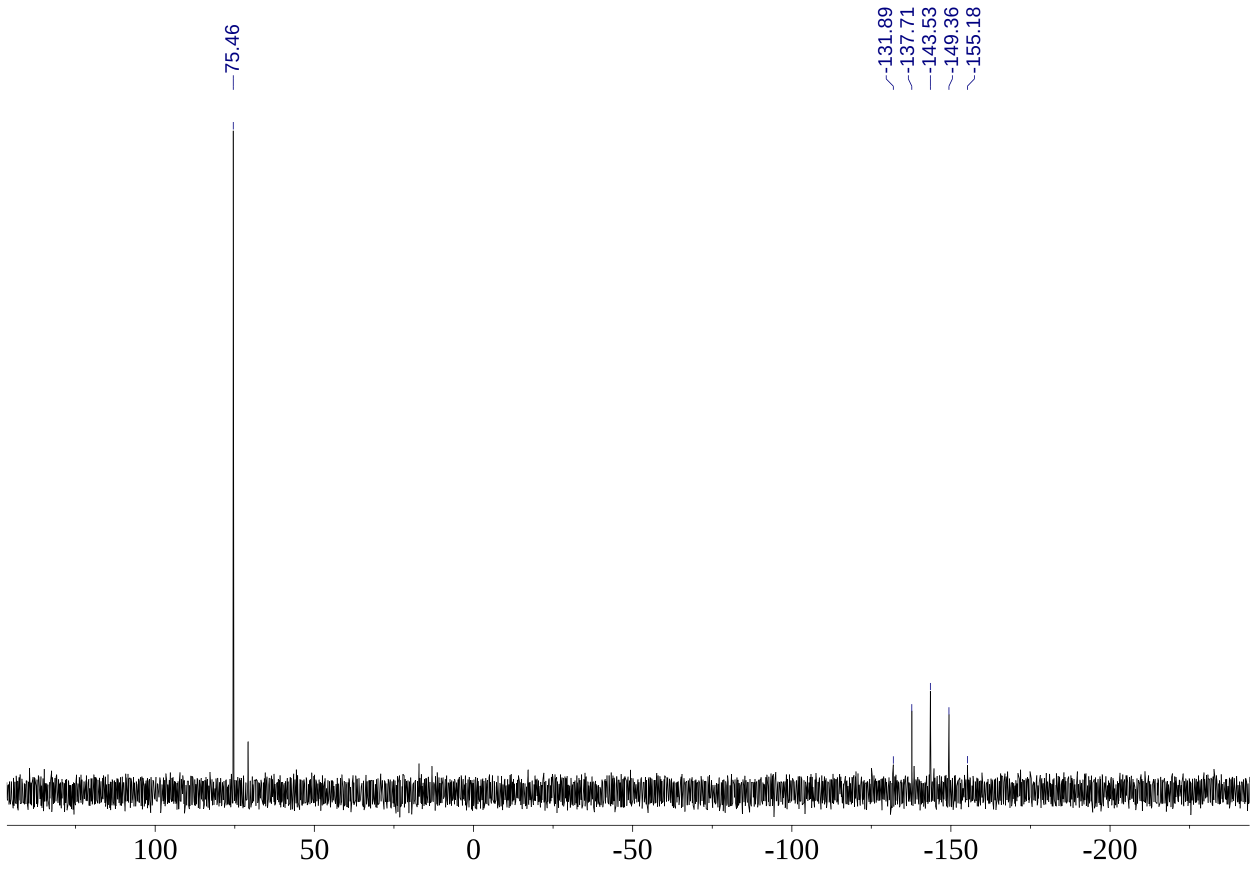

Figure S6. ^{31}P NMR spectrum of **2d** in acetone-d_6.

[Gold(III) 2-((diisopropylamino)(phenyl)phosphorothioyl)phenyl) (piperazin-1-dithiocarbamate)] hexafluorophosphate (2e)

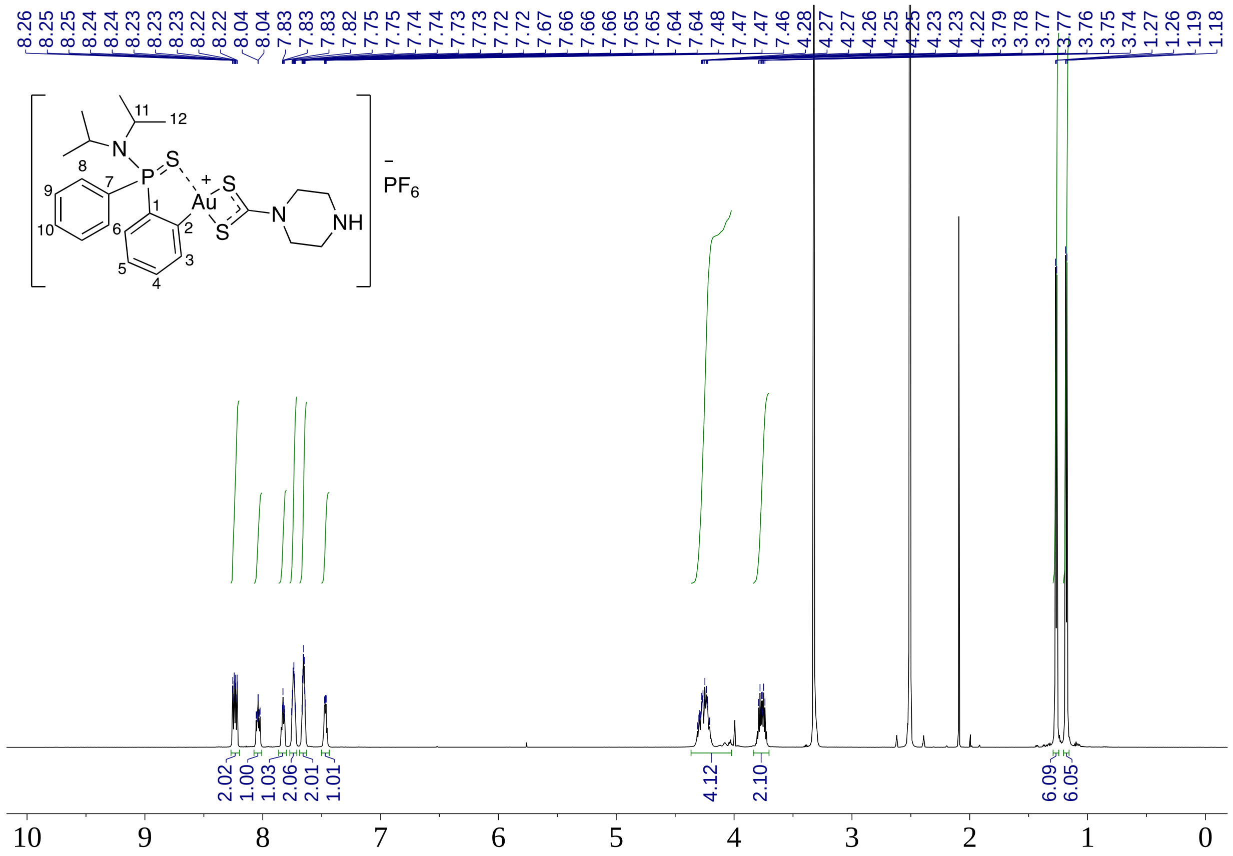

Figure S7. ^{1}H NMR spectrum of **2e** in $CDCl_3$.

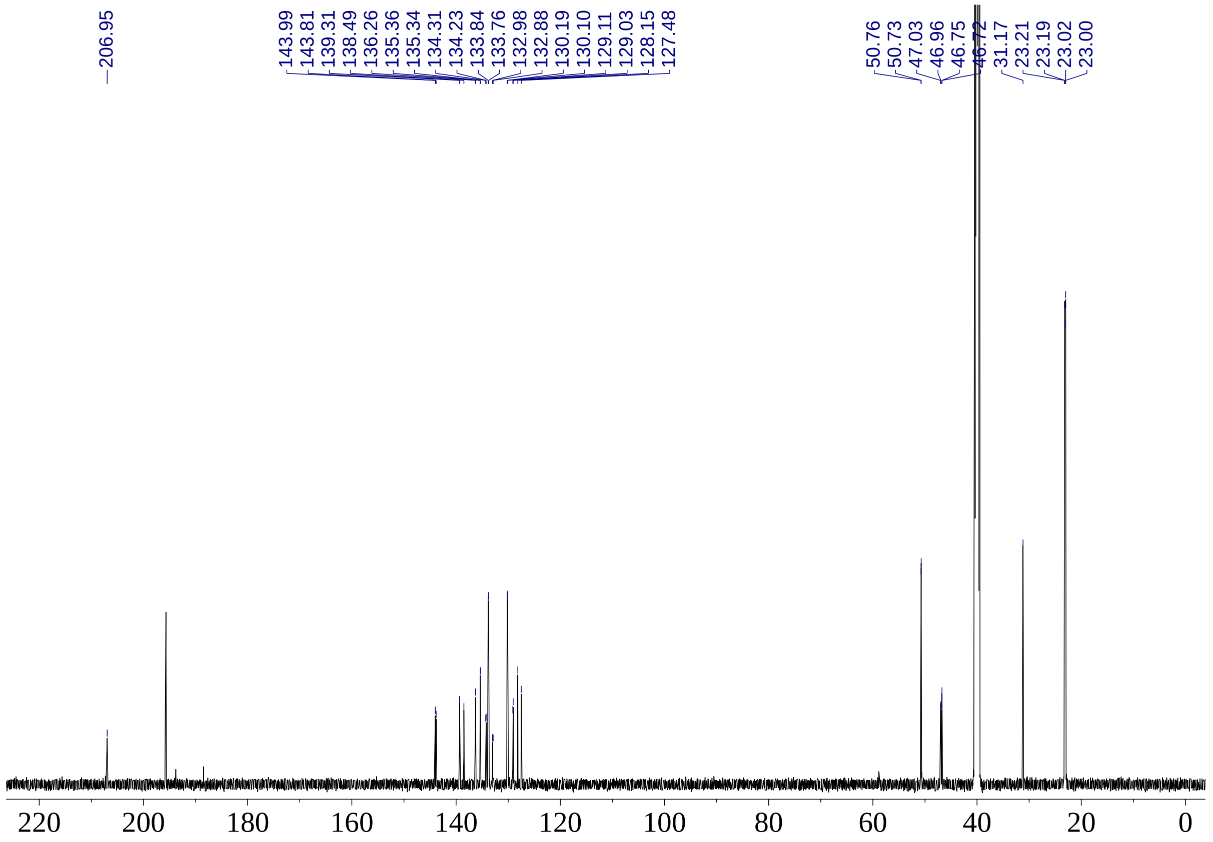

Figure S8. ^{13}C NMR spectrum of **2e** in $CDCl_3$.

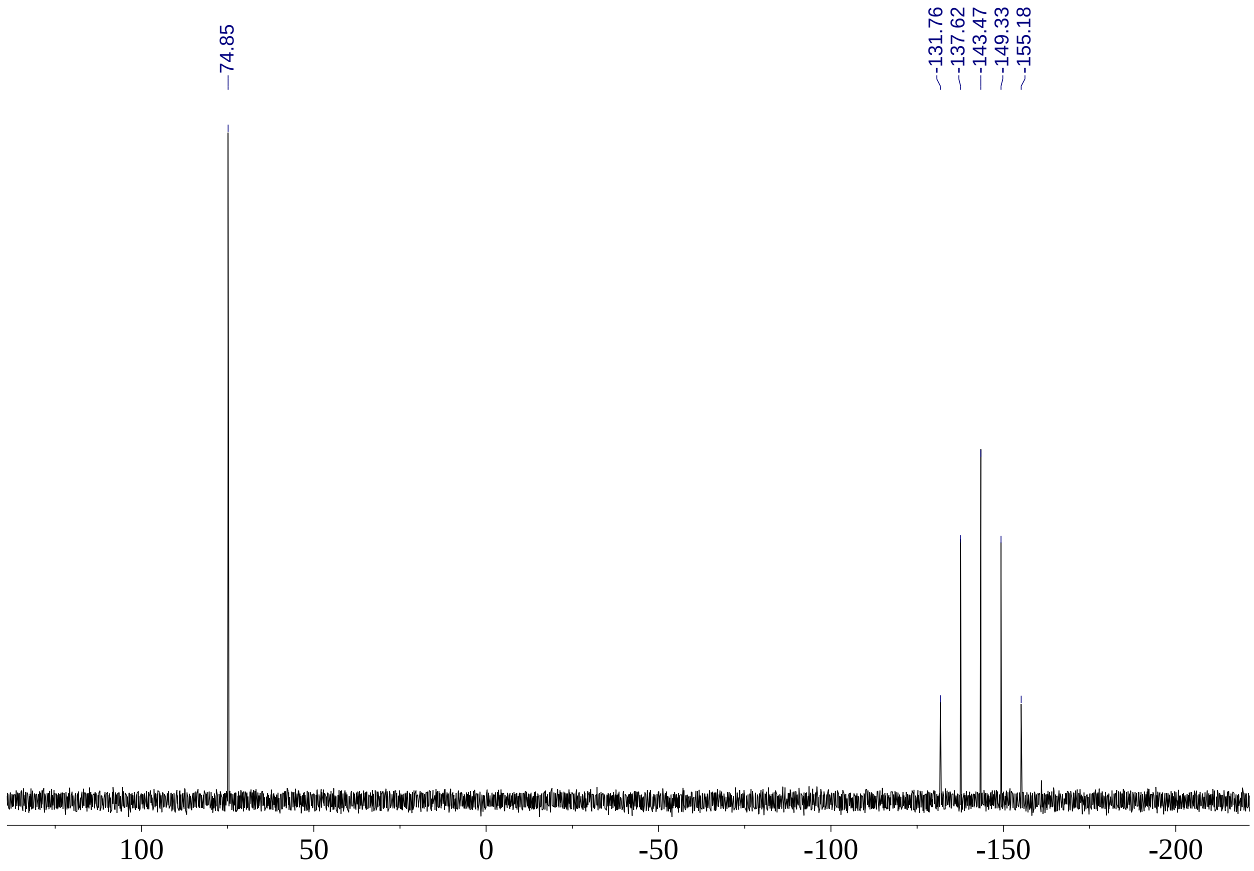

Figure S9. ^{31}P NMR spectrum of **2e** in $CDCl_3$.

[Gold(III) 2-((diisopropylamino)(phenyl)phosphorothioyl)phenyl) (azepan-1-dithiocarbamate)] hexafluorophosphate (2f)

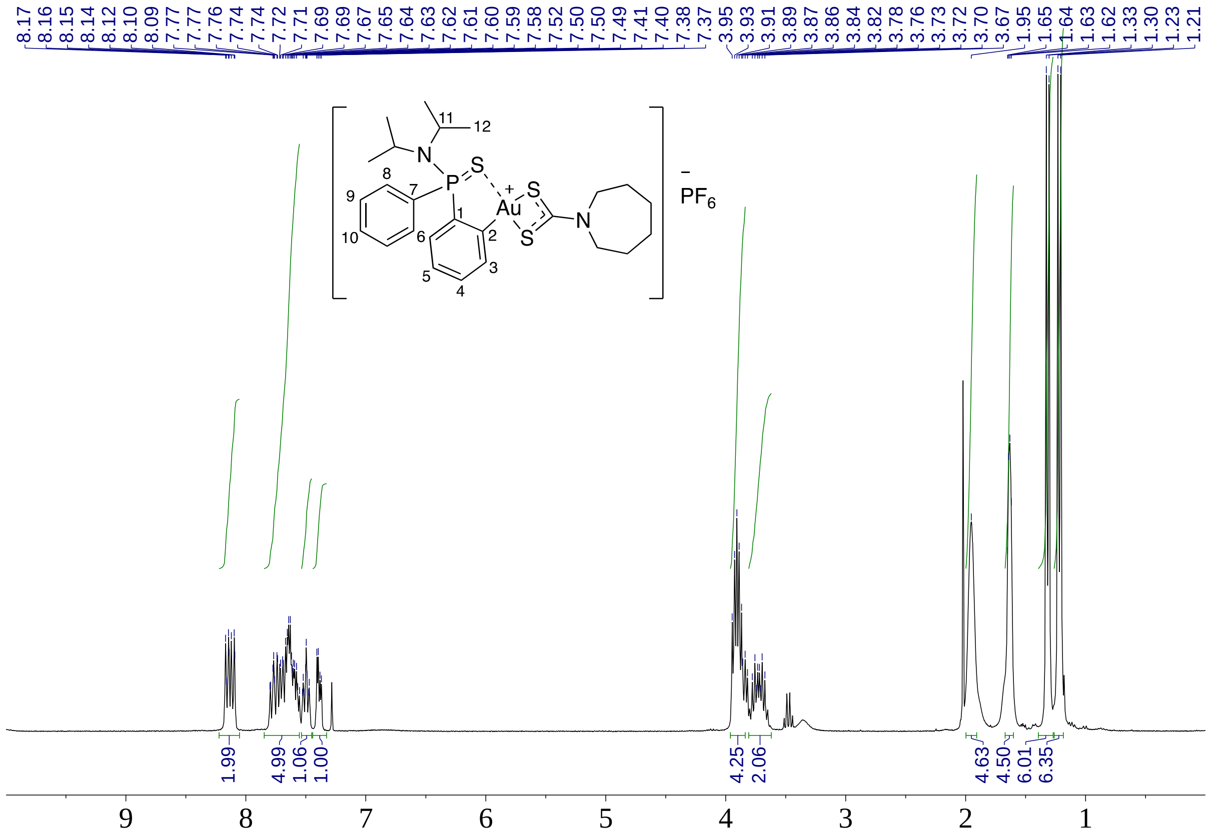

Figure S10. ^{1}H NMR spectrum of **2f** in $CDCl_3$.

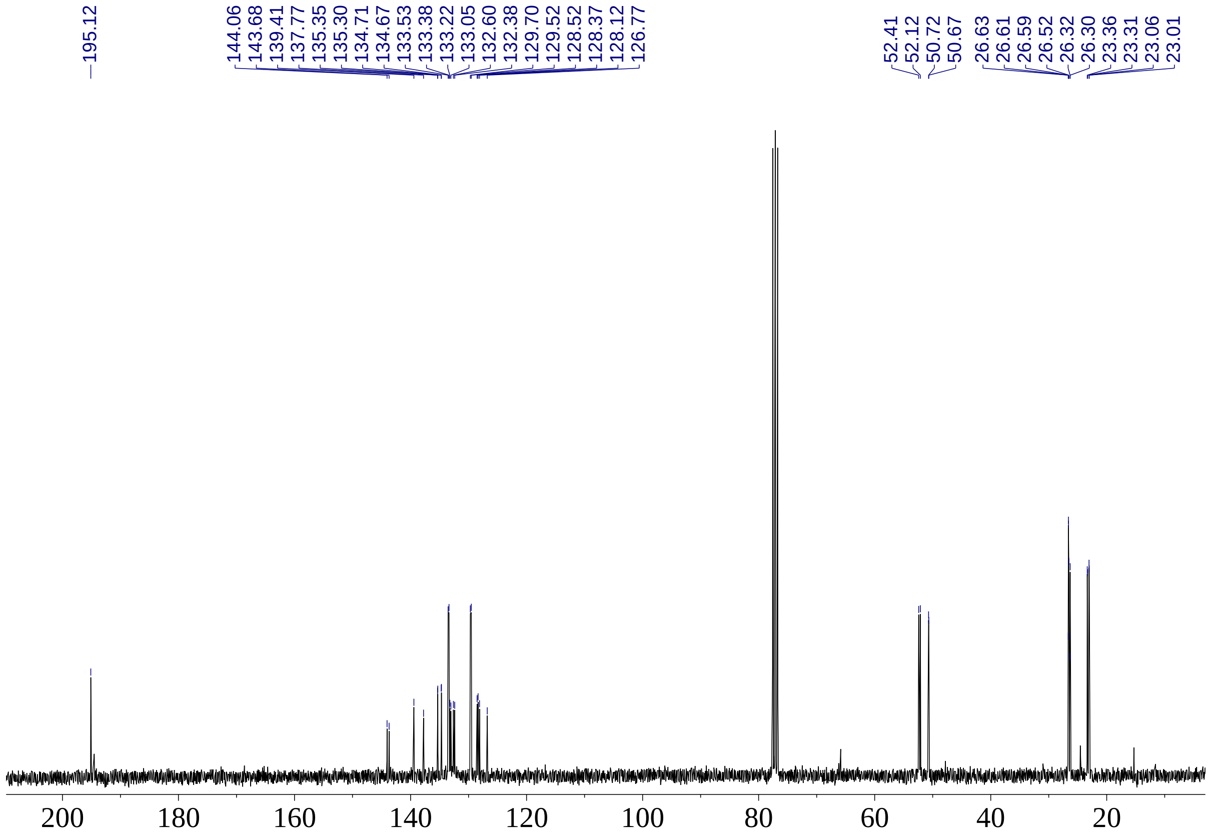

Figure S11. ^{13}C NMR spectrum of **2f** in $CDCl_3$.

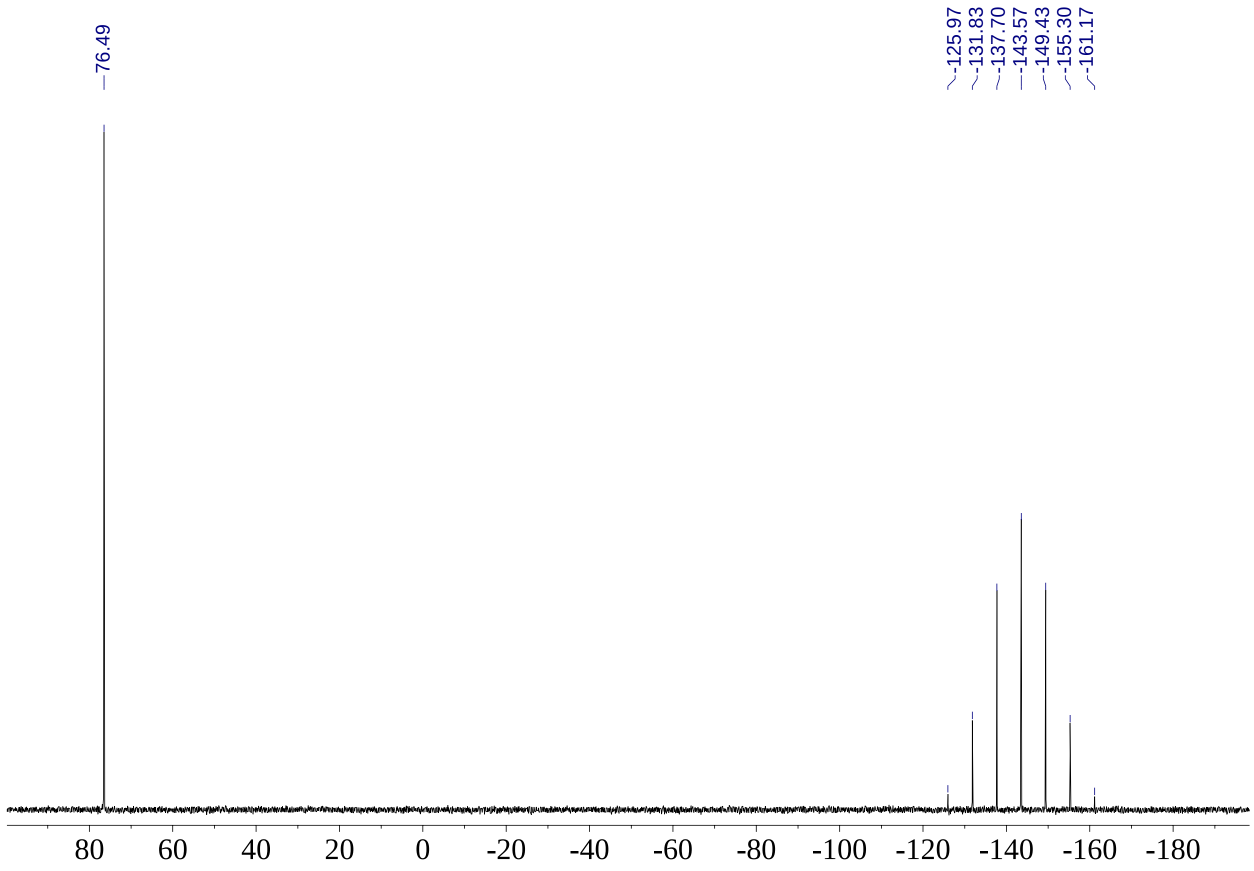

Figure S12. ^{31}P NMR spectrum of **2f** in $CDCl_3$.

[Gold(III) 2-(bis(diethylamino)phosphorothioyl)phenyl) (diethyldithiocarbamate)] hexafluorophosphate (3)

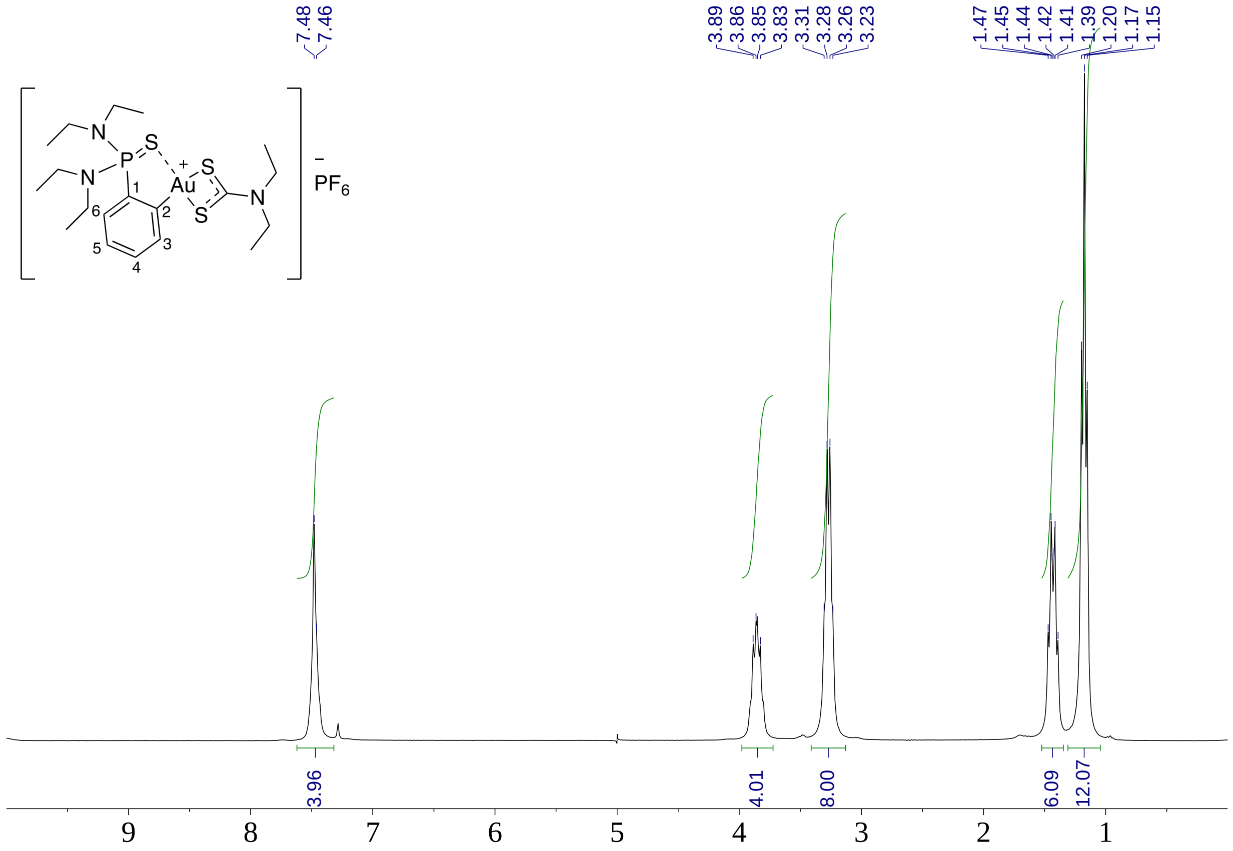

Figure S13. ^{1}H NMR spectrum of **3** in $CDCl_3$.

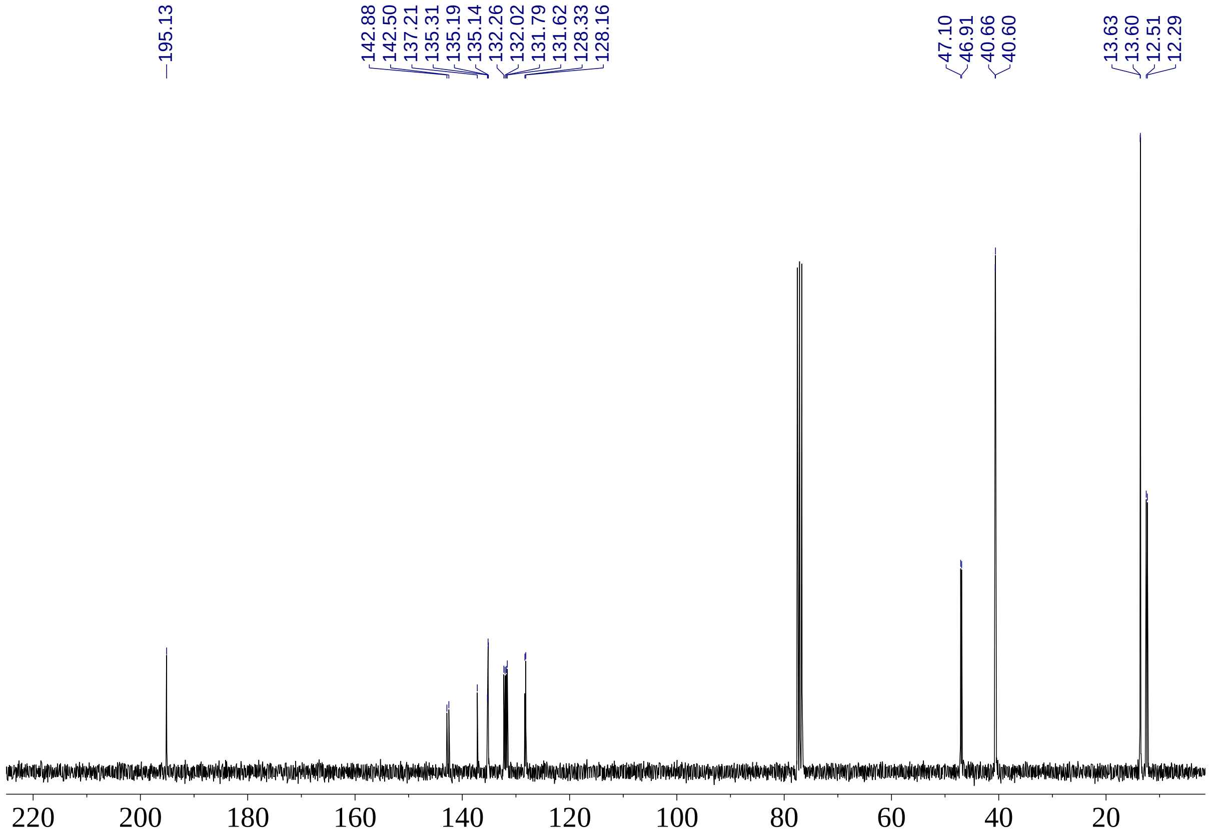

Figure S14. ^{13}C NMR spectrum of **3** in $CDCl_3$.

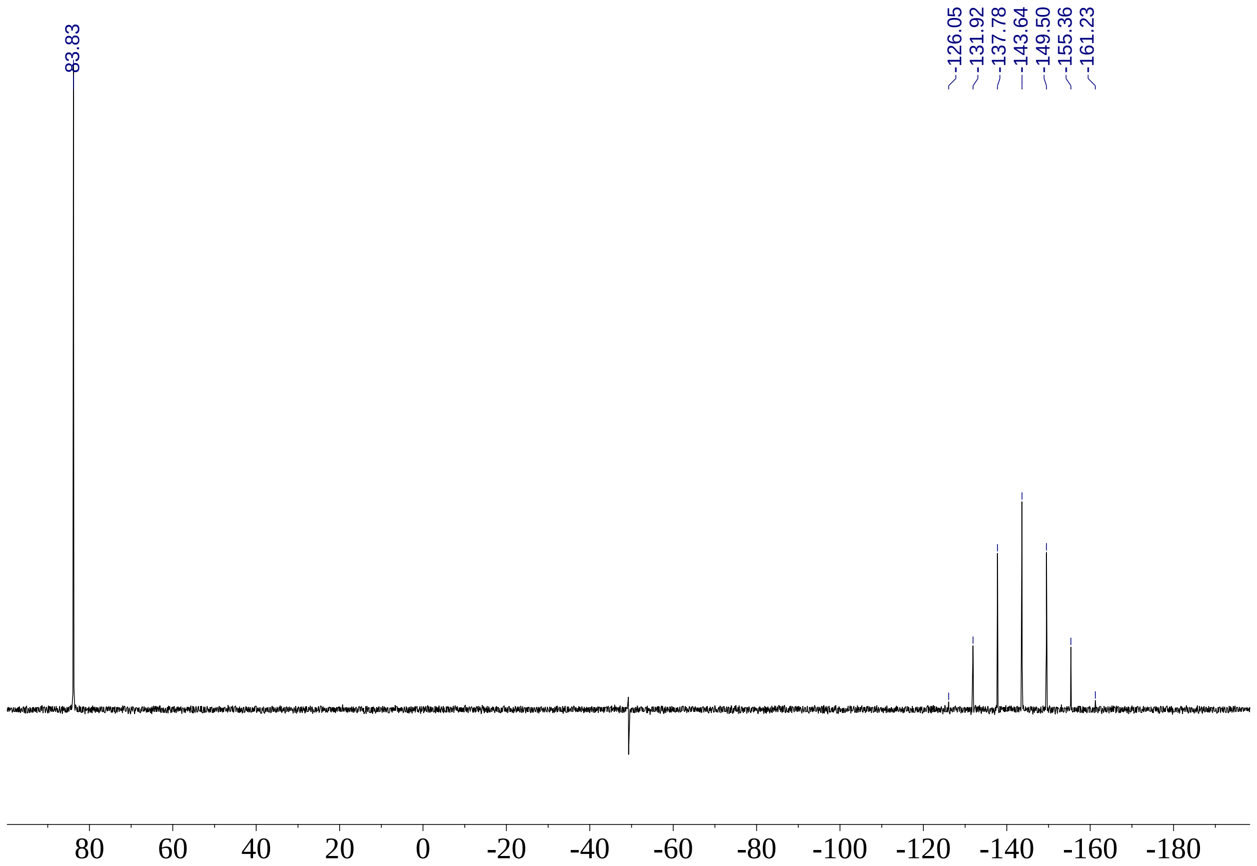

Figure S15. ^{31}P NMR spectrum of **3** in $CDCl_3$.
